# Antibiotic polymyxin arranges lipopolysaccharide into crystalline structures to solidify the bacterial membrane

**DOI:** 10.1038/s41467-022-33838-0

**Published:** 2022-10-21

**Authors:** Selen Manioglu, Seyed Majed Modaresi, Noah Ritzmann, Johannes Thoma, Sarah A. Overall, Alexander Harms, Gregory Upert, Anatol Luther, Alexander B. Barnes, Daniel Obrecht, Daniel J. Müller, Sebastian Hiller

**Affiliations:** 1 Department of Biosystems Science and Engineering, Eidgenössische Technische Hochschule (ETH) Zürich, Mattenstrasse 26, Basel, Switzerland; 2 Biozentrum, University of Basel, Spitalstrasse 41, Basel, Switzerland; 3 Department of Chemistry and Molecular Biology, University of Gothenburg, Göteborg, Sweden; 4 Laboratory of Physical Chemistry, ETH Zurich, Zurich, Switzerland; 5 Spexis AG, Allschwil, Switzerland; 6 Bachem AG, Bubendorf, Switzerland

**Keywords:** Antibiotics, Atomic force microscopy, Membrane structure and assembly, Lipopolysaccharides, Antimicrobial resistance

## Abstract

Polymyxins are last-resort antibiotics with potent activity against multi-drug resistant pathogens. They interact with lipopolysaccharide (LPS) in bacterial membranes, but mechanistic details at the molecular level remain unclear. Here, we characterize the interaction of polymyxins with native, LPS-containing outer membrane patches of *Escherichia coli* by high-resolution atomic force microscopy imaging, along with structural and biochemical assays. We find that polymyxins arrange LPS into hexagonal assemblies to form crystalline structures. Formation of the crystalline structures is correlated with the antibiotic activity, and absent in polymyxin-resistant strains. Crystal lattice parameters alter with variations of the LPS and polymyxin molecules. Quantitative measurements show that the crystalline structures decrease membrane thickness and increase membrane area as well as stiffness. Together, these findings suggest the formation of rigid LPS–polymyxin crystals and subsequent membrane disruption as the mechanism of polymyxin action and provide a benchmark for optimization and de novo design of LPS-targeting antimicrobials.

## Abstract (web summary)

Manioglu et al use high-resolution atomic force microscopy to resolve how polymyxins interact with the bacterial membrane. Polymyxins arrange the bacterial lipids into regular hexagonal structures that stiffen the membrane and lead to rupture.

## Introduction

Uncontrolled rise and spread of resistance in bacterial strains has transformed from individual observations in the 1940–1960s into a worldwide multi-drug resistance (MDR) crisis^1,2^, with substantial clinical and financial burden on healthcare systems^3^. Gram-negative bacteria are particularly progressive in developing resistance patterns, such that for MDR strains of *Enterobacteriaceae*, *Pseudomonas aeruginosa* and *Acinetobacter baumannii* the effective treatment options are limited^4^. Polymyxins are one of the few last-resort antibiotics effective against these pathogens^5,6^. They are biosynthesized as secondary metabolites of the Gram-positive bacterium *Paenibacillus polymyxa* and comprise of a cyclic heptapeptide attached to an *N*-terminal fatty acid tail through a tripeptide exocyclic linker^7^. Polymyxin E, also known as colistin, and polymyxin B are the two most studied and utilized variants. Amino acids are conserved among polymyxins, except for a hydrophobic residue at position six and variable fatty acid chains acetylated at position 1. Polymyxins were put into clinical use in the 1950s, but were subsequently replaced by other compounds due to nephrotoxic and neurotoxic side effects^8–10^. Nevertheless, improved dosing regimens and the rise of Gram-negative MDR strains led to a renaissance of their clinical use^5,11^.

Regarding their mechanism of action, there is general agreement that polymyxins function by interaction with lipopolysaccharide (LPS) molecules via their cationic l-α-γ-diaminobutyric acid (Dab) side chains and hydrophobic interactions of the residues at position 6 and 7^12–15^. LPS is composed of lipid A, a core oligosaccharide, and an O-antigen. LPS is synthesized in the bacterial inner membrane (IM) and then transported to the outer membrane via specific transport pathways, where it constitutes the dominant molecule of the outer leaflet^16–18^. The tight packing of LPS molecules is mediated by the divalent cations Mg^2+^ and Ca^2+^ that interact with the negatively charged lipid A moiety^19^. Notably, polymyxins interact with LPS in both the inner and the outer membrane (OM)^20^. The first interaction between the incoming antibiotic and LPS takes place in the OM. The interaction results in a mechanical alteration of the bacterial OM^21^, causing pronounced bulges on the bacterial surface and permeabilization of the OM^22^. Polymyxins then progress to the IM where they again interact with LPS molecules to eventually exert a lethal effect^20,23^.

The direct mechanism of interaction between LPS and polymyxin remains, however, unclear^24–26^. While it has been suggested that the membrane permeabilization might be caused by the fatty acyl tail of polymyxins in a detergent-like manner, recent experiments have questioned this, in particular, since the polymyxin concentrations reached in serum are not sufficiently high for such an effect^20,27,28^ and since polymyxins are specific for LPS^13,29^. In fact, the particularly low minimal inhibitory concentrations (MICs) in the low mg/l range featured by polymyxins make a structure-specific effect much more likely.

In this work, we study by atomic force microscopy (AFM) along with structural biology and biochemistry assays how the polymyxin antibiotics interact with LPS. AFM is a powerful tool for nanoscale exploration capable of working at different scales from cellular to membrane and even single molecule level^30^. Unlike other imaging techniques that require rough sample preparation or operation in non-physiological states, AFM can be used for imaging in a physiologically-relevant liquid environment^31^. Furthermore, AFM is capable of simultaneously imaging and quantifying the mechanical properties of cells, membranes, and molecules through its scanning force probe^32^. AFM can thus monitor structural changes while recording mechanical properties of bacterial membranes. For our studies, we utilize outer membrane vesicles (OMVs) of Gram-negative *E. coli* to form native membrane patches that contain LPS in the outer leaflet. Our experiments reveal hexagonal crystalline structures that form on OM patches upon polymyxin treatment. We characterize biophysical properties of these structures and correlate their appearance with the activity of polymyxins as well as the susceptibility of different bacterial strains. Our experiments provide evidence to suggest that the formation of crystalline structures constitutes the mechanism of action of polymyxins.

## Results

### Polymyxin induces crystalline structures in outer membranes

**Fig. 1:**
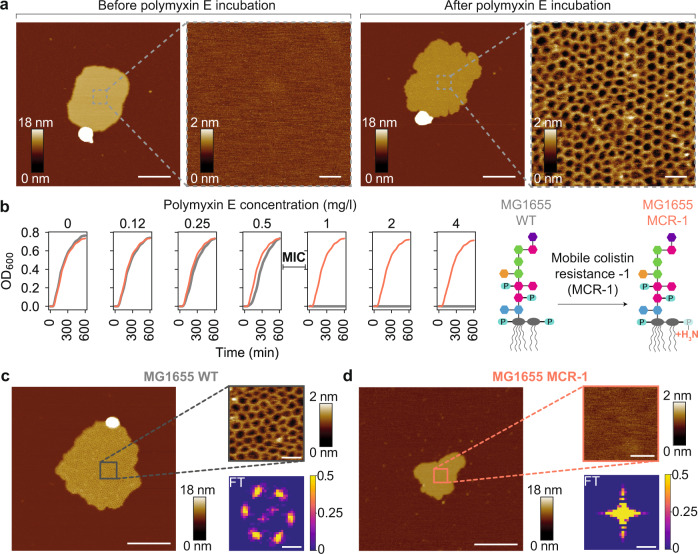
Polymyxin forms two-dimensional crystalline structures in bacterial outer membranes that correlate with antimicrobial activity. **a** AFM topographs of an OM patch before and after incubation with polymyxin E. Scale bars, 200 nm (overview) and 20 nm (zoom-in). **b** Growth curves of the *E. coli* MG1655 WT (gray) and MG1655 MCR-1 (red) strains at different polymyxin concentrations. Above the minimal inhibitory concentration (MIC) of 0.5–1 mg/l, the growth of the *E. coli* MG1655 WT strain is inhibited. Schematic of the effect of the MCR-1 modification on the LPS structures of the *E. coli* MG1655 strain. Source data are provided as a Source Data file. **c**, **d** AFM topographs (left) of OM patches from *E. coli* MG1655 WT and MCR-1 strains after polymyxin incubation around the MIC (0.6 mg/l). Right top, High-resolution images of framed areas. Scale bars, 200 nm (overviews) and 20 nm (insets). Right bottom, Fourier transformation of AFM images. The vertical scale represents image pixel intensity (a.u.). Each experiment was repeated independently at least three times with same results. Scale bars, 0.1 nm^−1^.

To study the effect of polymyxins on an LPS-containing membrane, OMVs were produced from the commonly used *E. coli* K-12 laboratory strain MG1655. OM hyper-vesiculation was stimulated by over-expression of mCherry in the periplasmic space (Supplementary Fig. 1a). The resulting OMVs were adsorbed on an atomically flat mica surface, where they opened as planar OM patches with diameters ranging from 100–300 nm. Topographies of the OM patches were recorded by AFM before and after incubation with polymyxins in imaging buffer (see Methods for details). Strikingly, incubation of the OM patches with 1 mg/l polymyxin E resulted in the formation of crystalline structures with a regular hexagonal lattice (Fig. 1a). The two-dimensional structures had a unit cell length of ≈ 10 nm and protruded ≈ 1–1.2 nm from the membrane surface. They formed with considerable long-range order, such that large crystalline arrays were observed in the membrane. Once the structures had formed, their shape and organization remained stable for several hours, as demonstrated by continuous imaging. The formation of a well-ordered structure readily suggested a role in polymyxin E function. We thus set out to identify characteristics as well as the occurrence of these structures.

As a first step, we probed the connection to polymyxin E antimicrobial activity. We measured the minimal inhibitory concentration (MIC) of polymyxin E on *E. coli* K-12 strain MG1655 by monitoring bacterial growth curves with increasing concentrations of the antibiotic. In our hands, the MIC of polymyxin E was 0.5–1 mg/l for *E. coli* MG1655, in agreement with published values^33^ (Fig. 1b). In parallel, we conducted AFM imaging at increasing polymyxin E concentrations and observed the formation of the crystalline structures initiated at a concentration matching the MIC (Fig. 1c). This direct correlation strengthens the hypothesis that the crystalline structures are involved in the mechanism of action of polymyxins. We then applied Fourier transform (FT) analysis on the AFM topographs, which resulted in a discrete diffraction pattern with hexagonal symmetry, directly indicating the presence of regular long-range order (Fig. 1c).

Next, we investigated OM patches from a polymyxin-resistant strain, where resistance was conferred by the mobile colistin resistance-1 (*mcr-1*) gene^25,34^. This gene encodes a membrane-associated enzyme that catalyzes a chemical modification of the lipid A phosphates by a phosphatidylethanolamine moiety (Supplementary Fig. 1b). The chemical modification reduces the net negative charge of the OM, thereby impairing the electrostatic interaction between lipid A and the cationic polymyxins^35^. In full agreement with the literature, *E. coli* MG1655 containing the MCR-1 plasmid^36^ (Supplementary Fig. 1c), was able to grow in considerably higher polymyxin E concentrations (32 mg/l) than the MG1655 wild-type (WT) strain (Fig. 1b). Plasmid-mediated expression of MCR-1 induced a substantial, population-wide change in size and morphology of the cells towards a non-native, spherical geometry that deviated strongly from the classical rod-like shape of *E. coli*, as evidenced by cryo-electron microscopy (cryo-EM) and fluorescence-activated cell sorting (FACS) (Supplementary Fig. 2a, b). Likewise, scanning electron microscopy (SEM) imaging demonstrated the differences in their surface texture with polymyxin E incubation. After polymyxin E treatment, the surface of the *E. coli* MG1655 WT strain exhibited bulges protruding from the bacterial surface, but MCR-1 bacteria did not exhibit this feature (Supplementary Fig. 2c). Strikingly, when we imaged OM patches prepared from the resistant strain by AFM, the supramolecular structures did not assemble, even at increased polymyxin E concentrations up to 600 mg/l (Fig. 1d, Supplementary Fig. 3). Correspondingly, FT of the OM patches did not generate a distinct pattern of spots, which confirmed the absence of a crystalline structure in these samples (Fig. 1d). Overall, the absence of crystalline structures in the resistant strain further strengthens a close relation between the crystalline structures and the antimicrobial activity of polymyxin E.

### LPS and divalent ions are essential for the formation of crystalline structures

Next, we wanted to characterize compositional requirements for the crystalline structures. To test for the necessity of LPS, we produced LPS-free lipid bilayer membranes from *E. coli* polar lipids. Upon incubation with 60 mg/l polymyxin E, no crystalline structures formed in these membranes, but solely a perturbation in the form of a lateral expansion of membrane bilayer was observed (Supplementary Fig. 4a). Then, to examine the necessity of proteins, we produced OMVs from the *E. coli* BL21(DE3) omp8 strain, which is deprived of the most abundant outer membrane proteins (OMPs)^37^, such that OMVs produced from this strain are largely devoid of OMPs^38^. This property allows the production of OMVs enriched with specific OMPs. Upon polymyxin E incubation, OM patches enriched in either OmpG or BamA exhibited similar supramolecular structures as membrane protein-devoid OM patches, which indicates that the crystalline structures form independently of the OM protein content (Supplementary Fig. 4b, c). Together, these observations suggest that the formation of the hexagonal crystalline structures requires LPS, but is not dependent on a specific membrane protein.

Next, we probed the role of divalent cations Ca^2+^ and Mg^2+^, which electrostatically bridge the LPS molecules of the OM (Supplementary Fig. 5). The crystalline structures formed also in the presence of high Mg^2+^ concentrations (5 mM), albeit at a slightly elevated polymyxin E concentration of 1.2 mg/l, suggesting that the crystalline structures are in competition with the native organization of LPS in the OM (Supplementary Fig. 5a). At the same time, however, divalent ions appear to be essential for the formation of crystalline structures, as the crystalline structures were fully disrupted upon treatment with the cation chelator ethylenediaminetetraacetic acid (EDTA) (Supplementary Fig. 5b). Similarly, pre-incubation of the OM patches with EDTA completely prevented the formation of the crystalline structures (Supplementary Fig. 5c). These observations thus suggest that divalent ions are involved in the formation of the crystalline structures, along with LPS and polymyxin E.

### Alterations in LPS length affect the structure dimensions

**Fig. 2:**
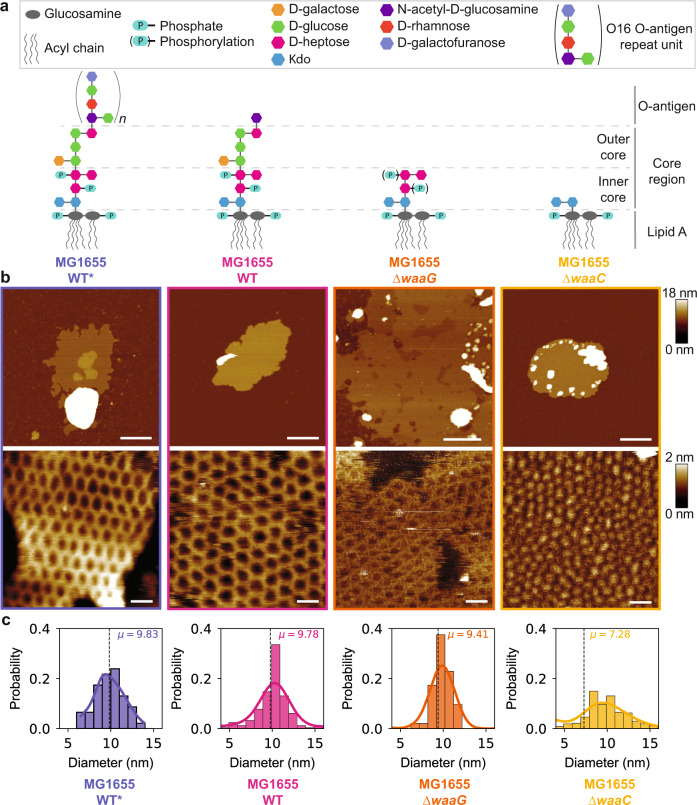
Structural parameters of crystalline structures respond directly to chemical alterations of the LPS molecule. **a** Schematic chemical structure of the LPS molecules in four different *E. coli* strains, MG1655 WT*, WT, ∆*waaG*, and ∆*waaC*. **b** Overview (top) and high-resolution (bottom) AFM topographs of OM patches from each *E. coli* strain, after incubation with polymyxin E. Scale bars, 200 nm (top panel) and 20 nm (bottom panel). **c** Histograms of the lattice constants of the two-dimensional crystalline structures and the mean ± standard deviation (SD) for analyzed structures (*n*): a_WT*_=9.8 ± 1.7 nm (*n* = 46), a_WT_ = 9.8 ± 3.9 nm (*n* = 244), a_Δ*waaG*_ = 9.4 ± 2.7 nm (*n* = 144), a_Δ*waaC*_ = 7.3 ± 3.7 nm (*n* = 367). Source data are provided as a Source Data file.

To establish a structure-activity relationship (SAR), we introduced structural variations both in LPS and polymyxin E and characterized the impact (Fig. 2). In order to investigate LPS-related effects, we generated OMV-producing bacterial strains with altered LPS lengths. The smooth LPS of the *E. coli* MG1655 WT* strain represents the wild-type situation of *E*. coli K-12 and is the longest LPS investigated in this study. This strain was prepared by repairing the *wbbL* gene that is inactivated in K-12 laboratory strains. Repair of this gene restores functional O-antigen expression and attachment to the core oligosaccharides^39^ (Supplementary Fig. 6a). We confirmed the presence of O-antigen with the binding of Atto 488-conjugated concanavalin A^40^ (Supplementary Fig. 6b, c). To obtain truncated LPS forms, we generated the *E. coli* MG1655 Δ*waaG* strain, resulting in an LPS molecule that lacks the outer core segment^18^. Lastly, the *E. coli* MG1655 Δ*waaC* strain has the shortest LPS lacking most of the inner core segment, because WaaC adds the first heptose to the 3-deoxy-D-manno-oct-2-ulosonic acid (Kdo) residue^18^. The Kdo, an eight-carbon sugar, connects the O-antigen and core domains to the lipid A moiety of LPS that is synthesized together with Kdo by a conserved biosynthetic pathway^18^.

Strikingly, the formation of supramolecular structures was observed for OM patches from each of these four strains, albeit with considerably modulated properties (Fig. 2a). In all cases, the assemblies on the OM patches exhibited a hexagonal lattice. High-resolution AFM images were analyzed quantitatively by an interactive machine learning software to determine the lattice constants a_WT*_, a_WT_, a_Δ*waaG*_, and a_Δ*waaC*_ of 9.8 ± 1.7 nm, 9.8 ± 3.9 nm, 9.4 ± 2.7 nm, and 7.3 ± 3.7 nm, respectively (Fig. 2b, Supplementary Fig. 7). The lattice constants of the crystalline structures reduced with the truncation of LPS length, confirming the involvement of this molecule. In addition to the lateral changes, we also observed distinct changes in the heights of crystalline structures. The shortest protrusions were exhibited by the *E. coli* MG1655 Δ*waaC* strain, which has the shortest LPS length in our library with only the inner core component. In addition, the long-range order of the hexagonal lattices broke down for this LPS variant. On the contrary, for the strains containing the outer core and extended components of the LPS, the long-range organization of the crystalline structures was preserved.

### Modifications of polymyxin affect the crystalline structures

**Fig. 3:**
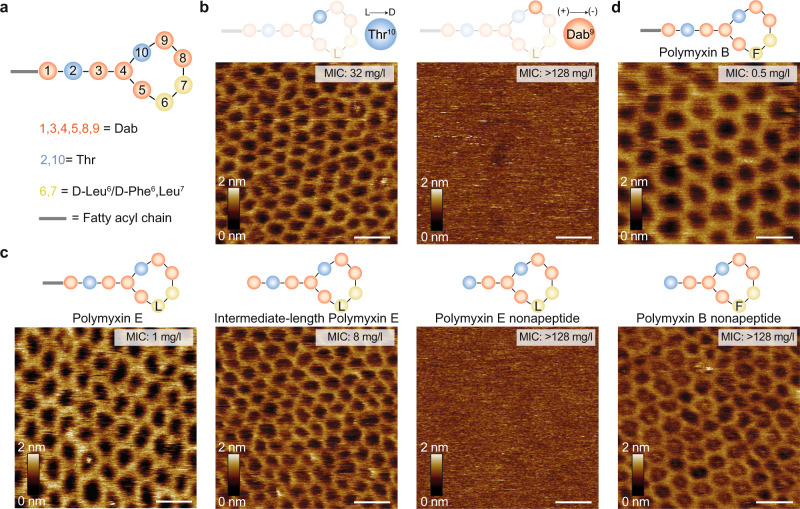
Changes in polymyxin composition affect the propensity to form crystalline structures. **a** Schematic representation of the polymyxin structure, where polymyxin E has D-Leu (L) and polymyxin B has D-Phe (F) in the 6^th^ position. **b**, **c** AFM topographs of OM patches from the *E. coli* MG1655 WT strain upon incubation with different polymyxin E variants. Crystalline structures are formed with the enantiomer variant Thr10 (L- > D) and with full and intermediate-length polymyxin E variants. Structures are not formed in the polymyxin variant Dab9 (+) -> (-) and polymyxin E nonapeptide variant. **d** AFM topographs of OM patches from the *E. coli* MG1655 WT strain upon incubation with polymyxin B and polymyxin B nonapeptide variants. Crystalline structures are formed in both cases. OM patches were incubated with 50 mg/l of the polymyxin variant. For **b**–**d**, each experiment was repeated independently at least three times with the same results. Scale bars, 20 nm.

Following our observations with altered LPS lengths, we also varied the structure of polymyxin (Fig. 3a, Supplementary Fig. 8a). Six out of the ten amino acid residues of polymyxins are cationic Dab residues resulting in a net positive charge at physiological pH^25^. Especially the Dab residues located in the heptapeptide ring are essential for the electrostatic interactions with the phosphate groups of lipid A^41^. Apart from the heptapeptide ring residues, previous studies have suggested the *N*-terminal fatty acyl chain to be crucial for antibacterial activity and toxicity of polymyxins^42,43^. Therefore, we decided to introduce chemical modifications in the ring and tail parts for studying their SAR. In the case of ring residue modifications, the polymyxin E analogue with a D-Thr10 residue induced the formation of crystalline structures on OM patches from the MG1655 WT strain. In contrast, the crystalline structures did not form, when Dab9 was changed to an azido moiety, thereby reducing the overall charge (Fig. 3b, Supplementary Fig. 8b).

To examine the effect of polymyxin backbone length on the formation of crystalline assemblies, we used three derivatives of polymyxin E (colistin). One was full-length polymyxin E, one intermediate variant lacked the *N*-termini fatty acyl chain, and one polymyxin E nonapeptide (PMEN) – lacked both the *N*-terminal fatty acyl chain and the cationic Dab1 residue (Fig. 3c, Supplementary Fig. 8c). AFM imaging of OM patches treated with either of these three polymyxin E derivatives showed that the full and intermediate variants induced formation of the crystalline structures, whereas the nonapeptide variant PMEN did not. We then also investigated full-length and nonapeptide variants of polymyxin B (PMB and PMBN, respectively), which both induced crystalline structures on OM patches upon incubation (Fig. 3d).

**Fig. 4:**
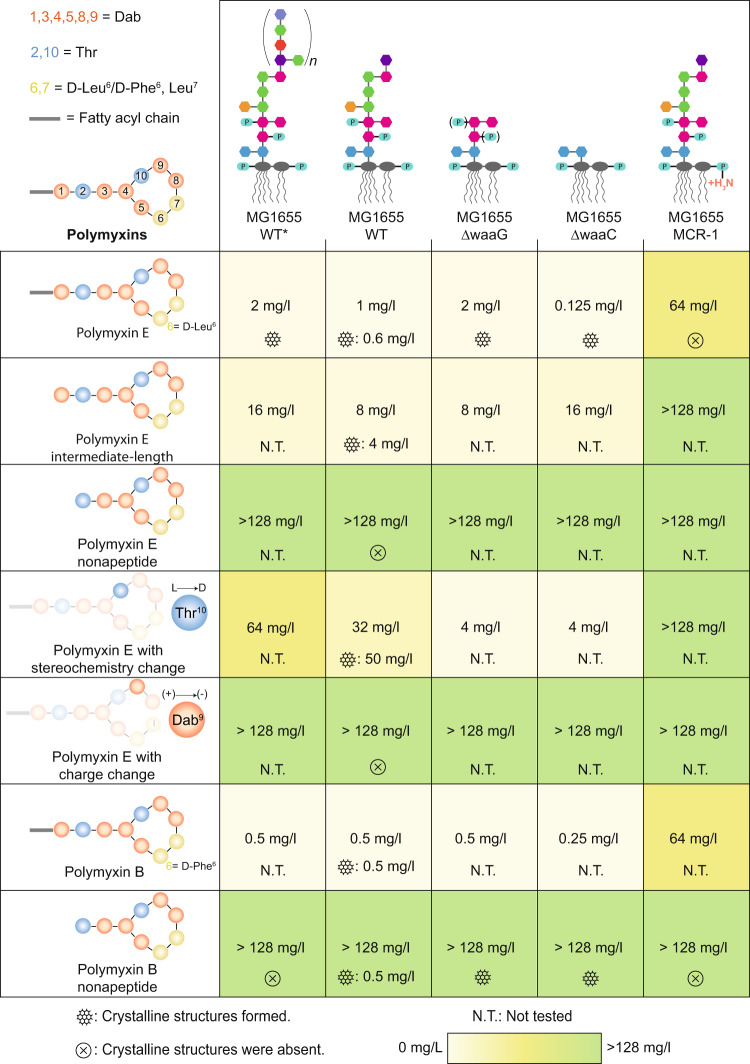
Minimal inhibitory concentrations (MICs) and crystalline structure formation propensity of polymyxin variants on different E. coli MG1655 strains. Each cell reports the MIC values as determined by an automated multi-well growth assay and the propensity of crystalline structure formation, along with the polymyxin concentrations that resulted in the formation of the crystalline structures. Cells are colored according to their MIC to guide the eye.

To get a clearer picture, we systematically determined the MIC values of seven different polymyxin variants with the five different *E. coli* MG1655 strains in an automated growth assay and compared with our observations on crystalline structure formation (Fig. 4). This extensive data set illustrates that the formation of crystalline structures is completely in line with the SAR of all variants of polymyxin E, as well as for polymyxin B. Furthermore, the MIC values are always close to the polymyxin concentrations required for inducing crystalline structure formation. The only outlier is presented by polymyxin B nonapeptide (PMBN), which appears to form crystalline structures despite a low antimicrobial activity. We thus conclude that the crystalline structures are necessary for the bactericidal activity of polymyxins. The particular behavior of PMBN can potentially be explained by the pivotal role of the D-Phe6 residue that in PMB has been identified to drive the hydrophobic parts of polymyxin towards the membrane^44,45^. It is well possible that the same interaction can facilitate the crystal formation of PMBN in the absence of a lipidic N-terminal tail. Overall, despite this peculiarity, the general correlation between MIC values and crystalline structure formation is apparent and is in full agreement with the known essentiality of the positively charged residues and the N-terminal fatty acid tail for the bactericidal activity of polymyxins^11,42^.

### Biophysical changes in the outer membrane upon polymyxin incubation

**Fig. 5:**
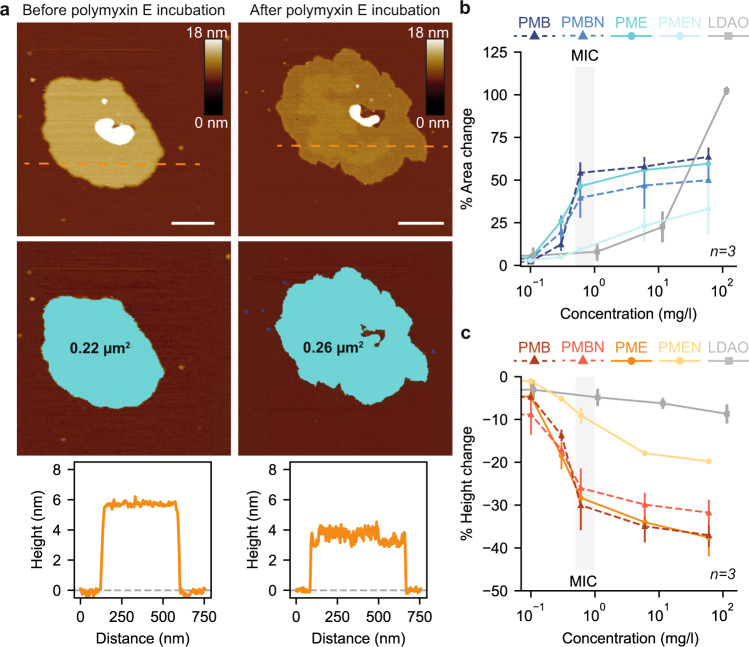
Polymyxin-induced formation of crystalline structures alters basic properties of the outer membrane. **a** AFM topographs (top) of an OM patch from the *E. coli* MG1655 strain before and after polymyxin E (1.3 mg/l) incubation for ~ 10 minutes with corresponding surface areas (middle) and height profiles (bottom) across the orange dashed lines. Scale bars, 100 nm. **b** Change in membrane area as a function of polymyxin E (PME), polymyxin E nonapeptide (PMEN), polymyxin B (PMB), polymyxin B nonapeptide (PMBN), and LDAO detergent concentration. **c** Change in membrane height as a function of polymyxin E (PME), polymyxin E nonapeptide (PMEN), polymyxin B (PMB), polymyxin B nonapeptide (PMBN), and LDAO detergent. For both experiments in panel **b** and **c**, the shaded area shows the MIC of polymyxin E. All conditions were measured for *n* = 3 OM patches from three independent experiments. Data points and error bars correspond to the mean and standard deviation, respectively. Source data are provided as a Source Data file.

Next, we characterized the effect of polymyxins on the biophysical properties of the OM (Fig. 5). Incubation with polymyxin E increased the membrane surface area and decreased the height (thickness) of the membrane (Fig. 5a). We monitored the relative changes at increasing concentrations (0, 0.1, 0.3, 0.6, 6, 60 mg/l) of either of the four compounds polymyxin E (PME), PMEN, PMB, and PMBN (Fig. 5b, c). Crystalline structures formed on OM patches upon incubation for PME, PMB, PMBN, and the changes in membrane area and height were overall similar for these three compounds. Remarkably, the alteration of membrane properties occurred in a non-linear fashion over the concentration range but appeared mainly as a single step at the concentration of crystalline structure formation. In stark contrast, the membrane area and height changes were considerably smaller for PMEN, in full agreement with its incapability to form crystalline structures on OM patches. Because the polymyxin mechanism of action has been suggested to be detergent-like in some publications, we also determined the effects with the zwitterionic detergent N, N-dimethyl-1-dodecanamine-N-oxide (LDAO), which acts on the OM in a non-specific manner (Supplementary Fig. 9a). Upon incubation with LDAO, the area of OM patches stayed constant for low and intermediate concentrations and dramatically increased by around two-fold at a detergent concentration of 11.5 mg/l, which is 20x times less than the critical micelle concentration (CMC) (Supplementary Fig. 9b, c). The height of the membrane remained roughly constant, with a maximal decrease of ≈ 9%, which is far less compared to the active polymyxin derivatives (Supplementary Fig. 9b, d). Thus, polymyxin crystalline structures have a dramatically different effect and concentration dependence on the OM than a common detergent. The crystalline structures expand the membrane laterally and make it thinner, whereas at comparable concentrations, the detergent leaves the area and thickness constant (Supplementary Fig. 9c–e). Importantly, the lateral expansion provides a direct rationale for the bulges protruding from polymyxin-treated bacteria, which can be understood as a deposit of excess lipids.

**Fig. 6:**
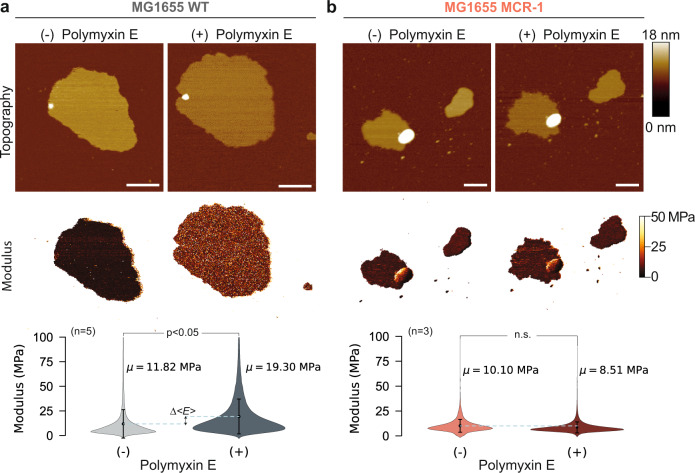
Bacterial membranes of E. coli MG1655 strain stiffen mechanically upon polymyxin incubation. **a** AFM topographs (top) and elastic modulus maps (middle) of an OM patch from the *E. coli* MG1655 strain before and after polymyxin E (60 mg/l) incubation for ~ 10 minutes. The elastic modulus of the patch increases upon polymyxin E treatment. The violin plots (bottom) show the modulus distribution of elastic modulus maps of OM patches (*n* = 5) before and after polymyxin E treatment. Dots indicate the mean modulus value, and vertical lines represent error bars as standard deviation. Difference between horizontal dashed lines (blue) denotes the positive increase in average Young’s modulus (Δ*<E* > ). Scale bars, 200 nm. **b** Same for an OM patch from an *E. coli* MG1655 MCR-1 strain. Scale bars, 200 nm. For both in panel A and B, nonparametric two-sided Mann-Whitney U test is used for comparing the modulus distributions before and after polymyxin E treatment. The change in the membrane modulus is significant for the *E. coli* MG1655 WT strain (*p* = 0.0079), whereas the change for the MCR-1 strain is insignificant (*p* = 0.4). Source data are provided as a Source Data file.

Next, we quantified the polymyxin-induced mechanical changes of the membrane. We employed multiparametric FD-based AFM to image the MG1655 OM patches incubated with polymyxin E to quantitatively map their elastic modulus and mechanical deformation. The data show that while the crystalline structures formed, the elastic modulus of the OM increased significantly (Fig. 6a). On the contrary, the elastic modulus of OM patches from the MG1655 MCR-1 resistant strain, displayed a slight but non-significant decrease upon incubation with polymyxin E (Fig. 6b). Further analysis on mechanical maps using topographical features showed that the modulus increase and deformation decrease coincide with the formation of crystalline structures in the membrane (Supplementary Fig. 10). To this end, we measured the gradual change in modulus and deformation through AFM imaging of OM patches with increasing concentrations of polymyxin E (Supplementary Fig. 10a). The crystalline structures approximately doubled the elastic modulus of OM patches and decreased their deformation by ≈ 20% (Supplementary Fig. 10b, c).

To corroborate the stiffness measurements with an orthogonal method, we employed in-cell solid-state NMR relaxation experiments on complete *E. coli* K-12 MG1655 bacteria in the absence and presence of polymyxin E. The spin relaxation parameter T_1_ρ, which measures the longitudinal relaxation of NMR spins in the rotating frame, probes structural fluctuations on the µs time scale and is a suitable parameter to assess membrane stiffness. The assignment of the 1D ^31^P-NMR spectrum was done as described before^46^. The ^31^P-NMR spectrum showed two dominant peaks, isotropic phosphate and the gel phase lipid (Supplementary Fig. 11a). The isotropic phosphate signal corresponds to mobile molecules such as free phosphate, sugar-phosphates, nucleic acids and other metabolites^47,48^. The increase in its T_1_ρ could indicate a reduced metabolic exchange or an increase in pH upon polymyxin treatment but is of little predictive power for the membrane effects of polymyxin. In contrast, the gel phase lipid peak corresponds to phosphate moieties in lipid membranes and is thus of particular interest in this study. T_1_ρ values determined for the gel phase lipid of *E. coli* strain MG1655 in the absence and presence of polymyxin E were equal to 2.6 and 4.0 ms, respectively. This increase suggests reduced dynamics of the gel phase lipid upon polymyxin E incubation. In agreement with this, the resistant MCR-1 strain did not respond with an increase in T_1_ρ to polymyxin E (Supplementary Fig. 11b–e). This negative control experiment was performed *n* = 1 times. The spectra show a notable difference in signal-to-noise ratio between MCR1 and WT, which is presumably due to a reduction in cross-polarization efficiency and differences in rotor packing due to the altered cell morphology. A titration experiment with increasing concentration of polymyxin E further confirmed the increase of rigidity of the gel phase lipid upon interaction with polymyxin E (Supplementary Fig. 11f, g). Overall, the data thus show that the formation of polymyxin–LPS crystalline structures is associated with a considerable increase in membrane stiffness.

## Discussion

**Fig. 7:**
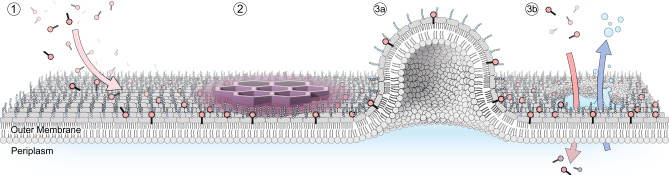
Model of the mechanism of action of polymyxins. **1** Polymyxins initially localize on the OM membrane of Gram-negative bacteria through electrostatic interactions with LPS. **2** On the membrane, polymyxins form hexagonal crystalline structures that increase the membrane surface area, decrease the membrane bilayer height, and stiffen the membrane. The altered mechanical properties of the OM lead to (**3a**) the formation of membrane bulging protrusions and (**3b**) membrane rupture, content leak, which allows progression to the inner membrane and subsequent bacterial death.

Polymyxins have been in active use for more than 70 years, and macroscopic effects on the bacterial outer membrane have been resolved by electron microscopy as early as 1969, at a resolution of tens to hundreds of nm. The AFM techniques employed in this work now uniquely improve the available resolution down to the nm scale and can thus resolve that polymyxin forms well-ordered hexagonal crystalline structures with LPS in membrane patches. The formation of crystalline structures is directly correlated to antibiotic activity, both in its concentration dependence and in its absolute occurrence. Furthermore, the SAR of their formation follows the SAR of polymyxin analogues in all cases except for PMBN (Fig. 4). Crystalline structure formation appears necessary, and in most cases, sufficient for bactericidal activity. Together, these observations highly suggest that the hexagonal crystals are a part of the polymyxin mechanism of action (Fig. 7).

Our experiments resolve that the hexagonal crystals consist of LPS, polymyxin, and divalent cations, however, the exact spatial arrangement of these molecules remains unclear at this point. The observation that the lattice constant and long-range order of the crystalline structures depend on LPS length suggests that a local ordering of LPS by polymyxin must lie at the core of the arrangement. Notably, the formation of a periodic LPS–polymyxin complex directly implies the presence of cooperative binding, which in turn explains the remarkably low MICs in the very low mg/l range. The natural compound polymyxin thus exploited an existing opportunity for a specific regular structure with LPS by evolution. The mechanism of action thus goes beyond a simple detergent-like effect as suggested previously^49^. Recent MD simulations of polymyxin-induced clusters^50,51^ have proposed an increase in the local order of LPS bilayers by crystal domains in hexagonal coordination, which was also found to be correlated with the bacterial sensitivity to antimicrobials^52,53^. The crystalline structures identified in this work likely give a rationale for these observations.

In addition to these microscopic connections to the polymyxin mechanism of action, the observation that the crystalline structures lead to an increase in macroscopic membrane stiffness associated with a significant lateral increase of the membrane area fits perfectly to the known phenotypes that polymyxin treatment induces bulge-like protrusions as a morphological change on the surface of intact bacteria^54^. These deformations appear in AFM studies as lipid clustering and formation of protrusions that create roughness on liposomes and bacterial membranes upon polymyxin treatment^13,22,55^. Our findings suggest that the bulges result as a consequence of the OM area increase, such that excess lipid is pushed into the bulges. Subsequently, these mechanical changes cause membrane stress which ultimately leads to its disruption.

It has been reported that subsequent to OM rupture, polymyxins interact also with the LPS of the IM, leading to its distortion^20^. While we can currently not study IM patches experimentally, due to the absence of suitable preparation protocols, it is tempting to speculate that the effect polymyxins have on the IM might resemble a combination of the detergent-like disruptions observed for *E. coli* polar lipid bilayers and the crystalline structures formed with LPS. The lateral expansions and stiffening would affect the integrity of the IM similarly to the OM and result in the cidal effect. The observations by NMR spectroscopy that polymyxin incubation increases the membrane rigidity in intact WT bacteria but not in the MCR-1 strain are in perfect agreement with this model.

Taken together, the findings presented in this work provide a mechanistic model for the mechanism of action of polymyxins at nanometer resolution. The understanding that polymyxins have been shaped by evolution to form a specific higher-order structure together with LPS, rather than engaging in a non-specific interaction with the membrane, will be essential for designing advanced variants of polymyxins to address the current antimicrobial resistance crisis.

## Methods

### Polymyxin variants

Polymyxin E, B, and B nonapeptide variants were purchased from Sigma-Aldrich, and the remaining polymyxin E variants are synthesized in-house by Spexis AG.

### Construction of plasmid pAH213_mcr1

In order to confer colistin resistance to the *E. coli* K-12 MG1655 strain and derivatives, an *mcr-1* gene together with its native promoter region was cloned onto the pBR322 plasmid backbone, creating pAH213_mcr1. Briefly, the *mcr-1* gene of colistin-resistant *E. coli* strain 2 HS-C was identified as gene E4F32_22195 on plasmid p2HS-C-1 (GenBank CP038181.1) and amplified together with ≈ 400 nucleotides of its immediate upstream region using primers prAH2159 and prAH2160. The backbone of pBR322 without its tetracycline resistance cassette was amplified using primers prAH1981 and prAH1982. Using 25 bp overhangs to the backbone included in prAH2159 / prAH2160, insert and backbone were ligated using Gibson assembly^56^. The resulting plasmid pAH213_mcr1 confers colistin resistance to the *E. coli* K-12 MG1655 strain with a minimal inhibitory concentration of 5 µg/ml (empty vector and vector-free controls: 0.5 µg/ml). Robust growth of the *E. coli* K-12 MG1655 strains carrying pAH213_mcr1 was observed up to a concentration of 3 µg/ml. The plasmids were transferred to the cells using the transformation and storage solution (TSS) method^57^.

### Engineering lipopolysaccharide (LPS) architecture

Derivatives of the *E. coli* K-12 MG1655 strain with altered LPS properties were used to study the effects of these alterations upon colistin treatment. In order to generate derivatives of the *E. coli* K-12 MG1655 strain with a semi-rough or rough phenotype, mutants lacking *waaG* or *waaC* were constructed by replacing the genes with a kanamycin resistance cassette for isogenic strains^58^. Expression of the O16-type O-antigen has been removed in the *E. coli* K-12 lineage of laboratory strains due to an insertion element disrupting the *wbbL* gene^39^. We restored the *wbbL* gene by deleting the insertion element using two-step recombineering as described^58^, creating the *E. coli* K-12 MG1655 *wbbL*(+) strain called *E. coli* MG1655 WT* throughout the manuscript. Combinations of LPS genotypes and different LPS modifications were achieved by transformation of these strains with plasmid pAH213_mcr1.

### Production of OMVs from different bacterial strains

*E. coli* MG1655 strains were transformed with the pY200 plasmid that carries an *mCherry* gene preceded by the periplasmic export signal of the skp protein under control of the *lux* promoter. This construct enables over-expression of mCherry into the periplasmic space, which in turn increases the OMV production. Cultures were grown in LB medium (Difco) with 50 µg/ml spectinomycin (Fischer Scientific) at 37 °C. Expression of mCherry was induced at an OD_600_ ≈ 0.4 by N-(β ketocaproyl)-L-homoserine lactone (HSL, Cayman Chemical). After induction, the cultures were grown until they reached the early stationary phase, and bacteria were removed by centrifugation at 10,000 *g* for 10 min. The supernatant was filtered through a 0.45 µm filter unit (Merck Millipore) for the removal of residual bacteria. The OMVs were collected from the filtered supernatant by centrifugation at 38,400 *g* for 1.5 h. The pellet was resuspended in 12 ml Dulbecco’s phosphate-buffered saline buffer supplemented with magnesium and calcium (Sigma-Aldrich), and collected by two rounds of ultracentrifugation at 100,000 *g* for 1 h each. In the last step, the OMVs were resuspended in 1 ml DPBS buffer, aliquoted, and stored at –80 °C.

### Preparation of *E. coli* polar lipid vesicles

*E. coli* polar lipid extract (Avanti Polar Lipids) in chloroform solution was transferred to a round-bottom flask. The chloroform was removed in a nitrogen stream by rotary evaporation to produce an even lipid film at the bottom of the flask. The lipid film was dried under vacuum in a desiccator overnight and rehydrated in DPBS buffer to a final lipid concentration of 1 mg/ml. The lipid suspension was passed through a 400 nm and a 200 nm NanoSizer MINI liposome extruder (T&T Scientific) to yield monodisperse vesicles.

### AFM imaging and analysis

OMVs produced from different *E. coli* MG1655 strains were adsorbed onto freshly cleaved mica for 15 min in DPBS buffer at room temperature. After adsorption, the sample was gently washed with fresh DPBS buffer for five times to remove non-adsorbed OMVs. Then OMVs were imaged using force-distance curve-based AFM (FD-based AFM) performed with an AFM (Nanoscope Multimode 8, Bruker) operated in PeakForce Tapping mode in buffer solution (DPBS) at room temperature^59^. The AFM was equipped with a 120 μm piezoelectric J scanner and fluid cell. The images were recorded using two different AFM cantilevers: PEAKFORCE‐HiRs‐F‐A (Bruker) with a nominal spring constant of 0.4 N/m, a resonance frequency of ≈ 165 kHz in liquid, and a sharpened silicon tip with a nominal radius of ≈ 1 nm or SCANASYST-FLUID + (Bruker) with a nominal spring constant of 0.7 N/m, a resonance frequency of ≈ 150 kHz in liquid, and a sharpened silicon tip with a nominal radius of ≈ 2 nm. Before imaging, cantilevers were calibrated by ramping on the mica surface and the thermal tuning method. Images were recorded at 2 kHz oscillation frequency, by applying an imaging force of 100–120 pN with a vertical amplitude of 30 nm. The AFM was placed inside a home‐built acoustic isolated and temperature‐controlled box. Raw AFM images were processed using the AFM analysis software Nanoscope v.1.8 for levelling and flattening.

### Fourier transformation of AFM images

AFM images were transformed with an automated image analysis script that identifies and characterizes local features by their Fourier transformation (FT)^60^. The analysis applies a moving local window (32×32 pixels, step size:16 pixels) on AFM images, and for each local window, a 2D power spectrum FT is computed, followed by machine learning processing. First, the number of components/fractions in the data is determined by computing the principal component analysis scree plot. Secondly, the data is blindly decomposed into different interpretable components by non-negative matrix factorization. The analysis script is publicly available as a Python analysis notebook and program for batch processing at (10.17632/25x46xjyr5.2).

### Parameter analysis of crystalline structures

Processed AFM images were analyzed using ilastik software (version 1.4.0b13) for automated (supervised) pixel classification with trained classifiers. Classifier files are available upon request. Through classification, the probability maps of AFM images were generated and exported as.tif files. The probability maps were opened in Fiji (ImageJ2 v.2.3.0) to perform particle analysis on threshold-selected features. The distance comparison histograms were generated from particle analysis data using Python’s data visualization library seaborn (v.0.11.2).

### Monitoring the growth curve of polymyxin incubated strains

All titration assays to measure the growth curve of *E. coli* MG1655 strains at different polymyxin concentrations were performed on a Tecan EVO 200 robotic platform in 96-well plates (cell culture microplates 96 well µClear® CELLSTAR®, Greiner Bio-One GmbH). Plates with serial dilutions of the tested polymyxin derivatives were prepared and overnight cultures of the strains were combined to a final OD_600_ ≈ 0.05. Titration culture plates were placed in a shaking platform equipped with a Kuhner ES-X shaking module (Adolf Kühner AG) mounted inside an aluminum housing (Tecan) and temperature-controlled using an “Icecube” (Life Imaging Services). Titration cultures were grown for 10 h at 37 °C with shaking at 300 rpm in clear-bottom 96-well plates, which were sealed with peelable foil with small slits (Agilent) to prevent liquid evaporation and guarantee sterility. OD_600_ measurements were recorded using a Tecan Infinite M200 Pro plater reader.

### Confocal imaging verification of O-antigen restored MG1655 WT* strain

Overnight cultures of *E. coli* MG1655 WT* and WT strains were inoculated to 2 ml fresh LB medium at a 1:200 ratio. The fresh cultures were grown at 37 °C with 220 rpm active shaking until they reached an OD_600_ of 0.5–0.6, and ODs were equilibrated before further steps. Cultures were transferred to 1.5 ml Eppendorf tubes and washed three times with DPBS buffer (1’000 µl). Cells were incubated with concanavalin A conjugated to Alexa Fluor 488 (Concanavalin A, succinylated, Alexa Fluor™ 488 conjugate, Invitrogen, Thermo Fischer Scientific) to a final concentration of 50 µg/ml at room temperature with 300 rpm shaking for labelling of the O16 antigen. After 1 h, cells were washed three times with DPBS buffer (1000 µl) to remove the excess labelling reagent. In the last step, cells were resuspended in DPBS buffer for fluorescence intensity examination with a Tecan M1000 plate reader and light microscopy. The epi-fluorescence images were collected with a Nikon Eclipse Ti2-E (inverted) equipped with Hamamatsu ORCA-Flash 4.0 camera. Samples were excited at 475 nm wavelength using a Lumencor Spectra X light engine, and the emission signal was collected at 525 nm with a Plan Apo λ 100x Oil Ph3 DM objective through a GFP_WF1 filter.

### Cryo-EM imaging of bacteria strains

Bacteria were grown as overnight cultures. Cells were transferred to a 5 ml tube and centrifuged for 4 min at 800 g. The supernatant was removed and the cell pellets were resuspended twice in 3 ml of Phosphate-buffered saline (PBS, Sigma-Aldrich). A sample with OD_600_ of 1.8 was then taken for vitrification. 5 µl of NiNTA-gold 10 nm as fiducial markers were mixed with the samples before vitrification. 4 µl of the samples were introduced to Lacey grids pre-treated with plasma under vacuum to make their surface hydrophilic. A Vitrobot FEI Eindhoven with 3 s of blot time standard and 1.0 blot force was used to vitrify the samples in liquid ethane. The imaging was performed using a 200 kV FEI Talos TEM equipped with a 16 M pixel CMOS camera.

### Fluorescence-activated cell sorting (FACS)

Samples for FACS were prepared by growing *E. coli* strain in LB media. Cultures were arrested by taking 12 h growing cultures into an ice slurry. FACS was done with a BD FACSAria III equipped with a blue, red, yellow-green, and violet laser operating at 488 nm, 561 nm, 633 nm, and 405 nm, respectively. FSC and SSC thresholds were set to 200 so that all cell events were detected and the electronic noise was reduced. In all experiments, 50’000 events were recorded at a low flow rate. The electronic abort rate was monitored carefully in all steps in order not to cross a 10% threshold rate.

### Solid-state NMR spectroscopy

Overnight cultures of the *E. coli* K-12 MG1655 strain were grown in Luria Broth containing 50 μg/ml spectinomycin and grown to OD_600_ of 1.5. For colistin treatment, 5 ml of bacteria in the stationary phase were treated with 0.5 μg/ml colistin for 2 h at room temperature and with 50 rpm shaking. The cells were then pelleted at 2’600 rpm for 5 min and gently washed with 5 ml phosphate-free NMR buffer (50 mM PIPES, pH 7.4, and 50 mM NaCl). The cell pellet was transferred to a custom-made Teflon funnel fitted to a 3.2 mm regular walled ZrO_2_ Bruker rotor (30 μl sample volume) and centrifuged for 30 s at 2500 *g*.

Static ^31^P-NMR spectra were acquired on a Bruker 11.7 Tesla NMR spectrometer operating at 500 MHz ^1^H Larmor frequency equipped with a Bruker 3.2 mm HXY MAS probe. ^31^P detected experiments were acquired with a ^31^P 90 pulse of 5 μs (50 kHz), recycle delay of 3.5 s, and under 85.3 kHz ^1^H spinal64 decoupling. 256 transients were collected with a recycle delay of 5 s. ^31^P chemical shifts were externally referenced to 85% H_3_PO_4_. T_1_ρ experiments were conducted with a ^31^P direct excitation 90° pulse of 3.5 μs (71.5 kHz) and ^31^P spin-locking field of 30 kHz with 83.5 kHz ^1^H spinal64 decoupling during spin-locking and acquisition. T_1_ρ relaxation data was collected by varying the spin-locking pulse width from 100 μs to 25 ms. 448 transients were collected per pulse width measured. The total acquisition time was 5 h per sample. Spectra were processed in Topspin 4.07 with a shifted sine bell function and 10 Hz line broadening. Measured intensities from peaks at 1 ppm and –11.3 ppm were fitted to a single exponential decay in GraphPad Prism to extract T_1_ρ values. To limit cellular changes over the acquisition period, all experiments were performed at 280 K.

### Reporting summary

Further information on research design is available in the Nature Research Reporting Summary linked to this article.

## Supplementary information

Supplementary Information

Reporting Summary

## Data Availability

The experimental data that support the findings of this study is available within this article or its supplementary materials. The data that support this study are available from the corresponding authors upon reasonable request. The datasets for lattice constant, biophysical change and modulus map analysis are available in the Zenodo repository, [10.5281/zenodo.6762552], and is publicly available as of the date of publication (MuellerLabETHZ, 2022). The source data underlying Figs. 1b, 2c, 5, and Supplementary Figs. 6b, 9b-e, 10b-c, 11 are provided as a Source Data file. Source data are provided with this paper.

## Source data

Source Data

## Code availability

Data analysis and visualization scripts used in this study are publicly available in the Zenodo repository, [10.5281/zenodo.6762552], and is publicly available as of the date of publication (MuellerLabETHZ, 2022).
