# Supplementary material for: Antibiotic polymyxin arranges lipopolysaccharide into crystalline structures to solidify the bacterial membrane
Source: Nat Commun. 2022 Oct 21;13:6195. doi: 10.1038/s41467-022-33838-0 (PMC9587031; doi:10.1038/s41467-022-33838-0)
Supplement: Supplementary file 1 — Supplementary Information [file 41467_2022_33838_MOESM1_ESM.pdf]

Supplementary Information

Antibiotic polymyxin arranges lipopolysaccharide into crystalline structures to solidify the bacterial membrane

Selen Manioglu, Seyed Majed Modaresi, Noah Ritzmann, Johannes Thoma, Sarah A. Overall, Alexander Harms, Gregory Upert, Anatol Luther, Alexander B. Barnes, Daniel Obrecht, Daniel J. Müller, Sebastian Hiller

This PDF file contains:

- **Supplementary Table 1 – 3**
- **Supplementary Fig. 1 – 10**

Supplementary Tables

Supplementary Table 1. List of bacterial strains used in this study.

Strain #	Strain name	Genotype	Resistance against polymyxin	Plasmid carried	Resistance from plasmid	Notation in this study
**1**	*E. coli* K-12 MG1655	*F- λ- ilvG-rfb-50 rph-1*	-	pY200 - mCherry	Spectinomycin	MG1655 WT
**2**	*E. coli* K-12 MG1655	*F- λ- ilvG-rfb-50 rph-1*	mcr-1.1	pY200 - mCherry and pAH213 - mcr-1.1	Spectinomycin, Polymyxin, Ampicillin	MG1655 MCR-1
**3**	*E. coli* K-12 MG1655 Δ*waaC*[1]	MG1655 *waaC::kanR*	-	pY200 - mCherry	Spectinomycin	MG1655 Δ*waaC*
**4**	*E. coli* K-12 MG1655 Δ*waaG*[1]	MG1655 *waaC::kanR*	-	pY200 - mCherry	Spectinomycin	MG1655 Δ*waaG*
**5**	*E. coli* K-12 MG1655 *wbbL(+)*	MG1655 *wbbL*(+)	-	pY200 - mCherry	Spectinomycin	MG1655 WT*
**6**	*E. coli* 2 HS-C[2]	*n.a.*	mcr-1.1; pmrB: H2R	-	-	
**7**	*E. coli* BL21(DE3)*omp8*[3]	BL21(DE3), *ΔlamB ompF Tn5 ompA ompC*	-	pY27	Ampicillin	OmpG
**8**	*E. coli* BL21(DE3)*omp8*[3]	BL21(DE3), *ΔlamB ompF Tn5 ompA ompC*	-	pY161	Ampicillin	BamA

Supplementary Table 2. List of oligonucleotide primers used in this study.

Primer	Oligonucleotide sequences (5'-3')
**prAH2159**	GAATTCTCATGTTTGACAGCTTATCGCGAACCTTATTACTTATTTTGGA
**prAH2160**	CGCTTCGTTAATACAGATGTAGGTGAAAAAATACAGGGAGAAATCAGC
**prAH1981**	CACCTACATCTGTATTAACGAAGC
**prAH1982**	GATAAGCTGTCAAACATGAGAATTC
**prAH2413**	CTGCAGCTAATAACCACTTTTTC
**prAH2414**	GTTATGCCCGTTTCAACATC
**prAH2463**	ATGGCGATGAAAAAGTTGCT
**prAH2464**	TTACCAGGTTTTACCGATGTTAAAC
**prAH2471**	AAACCGTCTGTACCATAACTCTTGTTGGTATAGTG
**prAH2472**	AAAACACTATACCAACAAGAGTTATGGTACAGACG
**prAH2473**	AAACAGAGATGCCGTTCTACGAGAACTTCTATGCG
**prAH2474**	AAAACGCATAGAAGTTCTCGTAGAACGGCATCTCT
**prAH2489**	AAACAGAGTACAGATAACGCCACATCGCAACCTGG
**prAH2490**	AAAACCAGGTTGCGATGTGGCGTTATCTGTACTCT

Supplementary Table 3. List of plasmids used in this study

Plasmid	Encoded gene	Resistance
**pY200**	mCherry	Spectinomycin
**pAH213_mcr1**	mcr-1	Polymyxin, Ampicillin
**pY27**[4]	OmpG	Ampicillin
**pY161**[4]	BamA	Ampicillin

Supplementary Figures

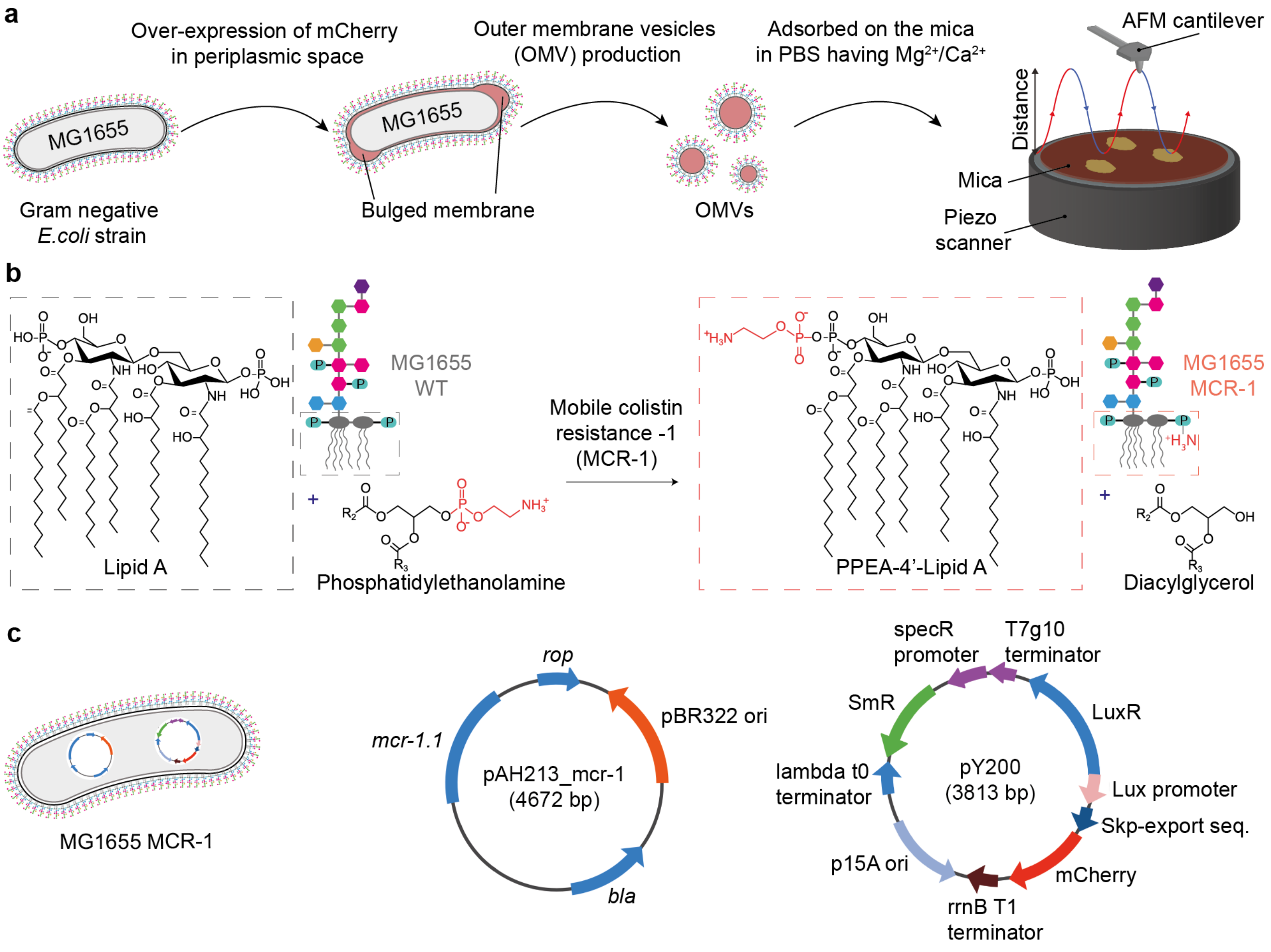

Supplementary Fig. 1. Generation of the *E. coli* MG1655 MCR-1.

(a) Production of OMVs from *E. coli* MG1655 strain and their adsorption on the mica surface in Dulbecco's phosphate-buffered saline (DPBS) buffer for AFM imaging. **(b)** Schematic of the effect of the plasmid-borne mobile colistin resistance-1 (*mcr-1*) gene on the lipid A moiety of the LPS. 4-phosphoethanolamine (PEA) is added to lipid A at the 4'-phosphate group, resulting in a decreased net negative charge, reducing the affinity to polymyxin. **(c)** Maps of plasmids transformed into the *E. coli* MG1655 strain. pAH213_mcr-1 confers colistin resistance and pY200 induces OMV hypervesiculation *via* periplasmatic mCherry expression.

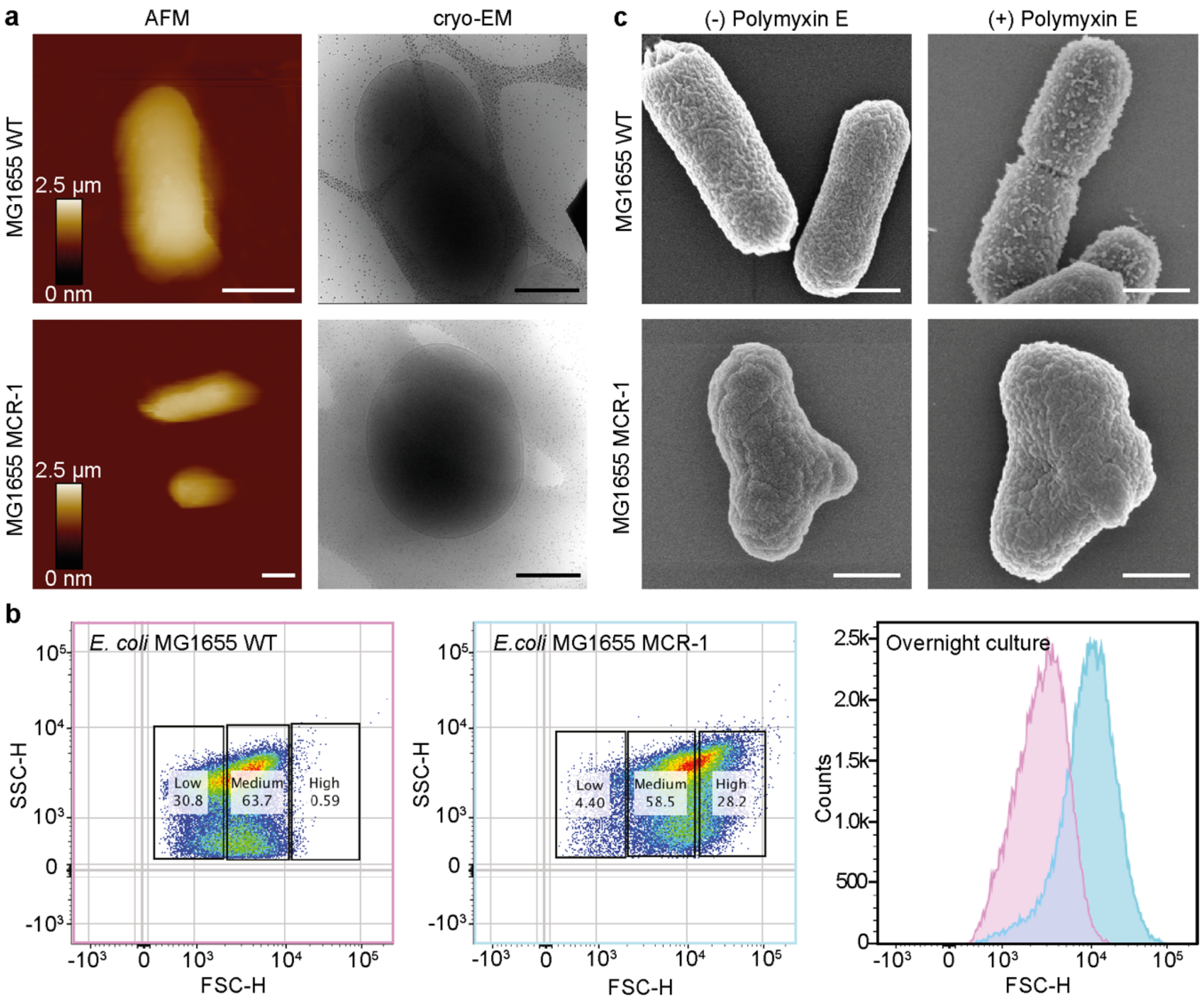

Supplementary Fig. 2. MCR-1 expression confers sphericity to *E. coli* morphology.

(a) AFM topography and cryo-EM images of rod-shaped like MG1655 WT *E. coli* strain (top) and circular-like MCR-1 *E. coli* strain (bottom). Scale bars are 1 µm for AFM topographs and 500 nm for cryo-EM images. AFM topographs were taken in DPBS buffer. **(b)** Fluorescence-activated cell sorting (FACS) data of MG1655 WT and MCR-1 *E. coli* strains for long culturing times. **(c)** Scanning electron microscopy (SEM) images of MG1655 WT and MCR-1 *E. coli* strains in the absence and presence (8 mg/l) of polymyxin. In the case of the MG1655 WT strain, polymyxin induces the formation of bulges on the bacterial surface, whereas the MG1655 MCR-1 strain lacks the appearance of similar structures. For a and c, each experiment was repeated independently two times with same results. Scale bars, 500 nm.

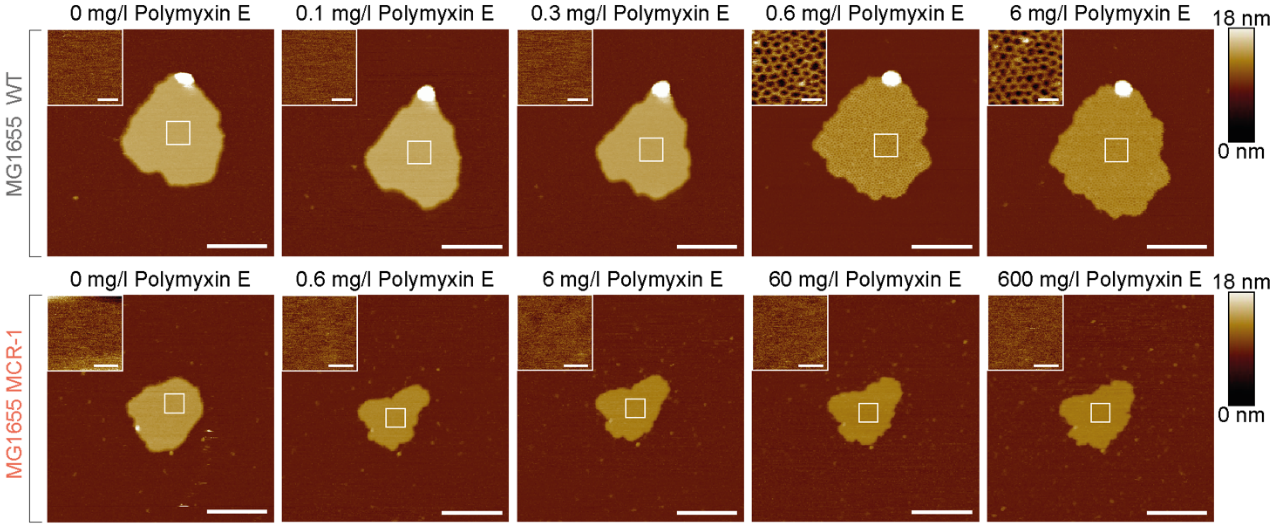

Supplementary Fig. 3. Monitoring the occurrence of crystalline structures on OM patches while ramping the polymyxin concentration.

AFM topographs of OM patches produced from MG1655 WT (top) and MCR-1 (bottom) strains incubated with increasing concentrations of polymyxin in DPBS buffer. Each experiment was repeated independently at least three times with same results. Scale bars, 200 nm and 20 nm (insets).

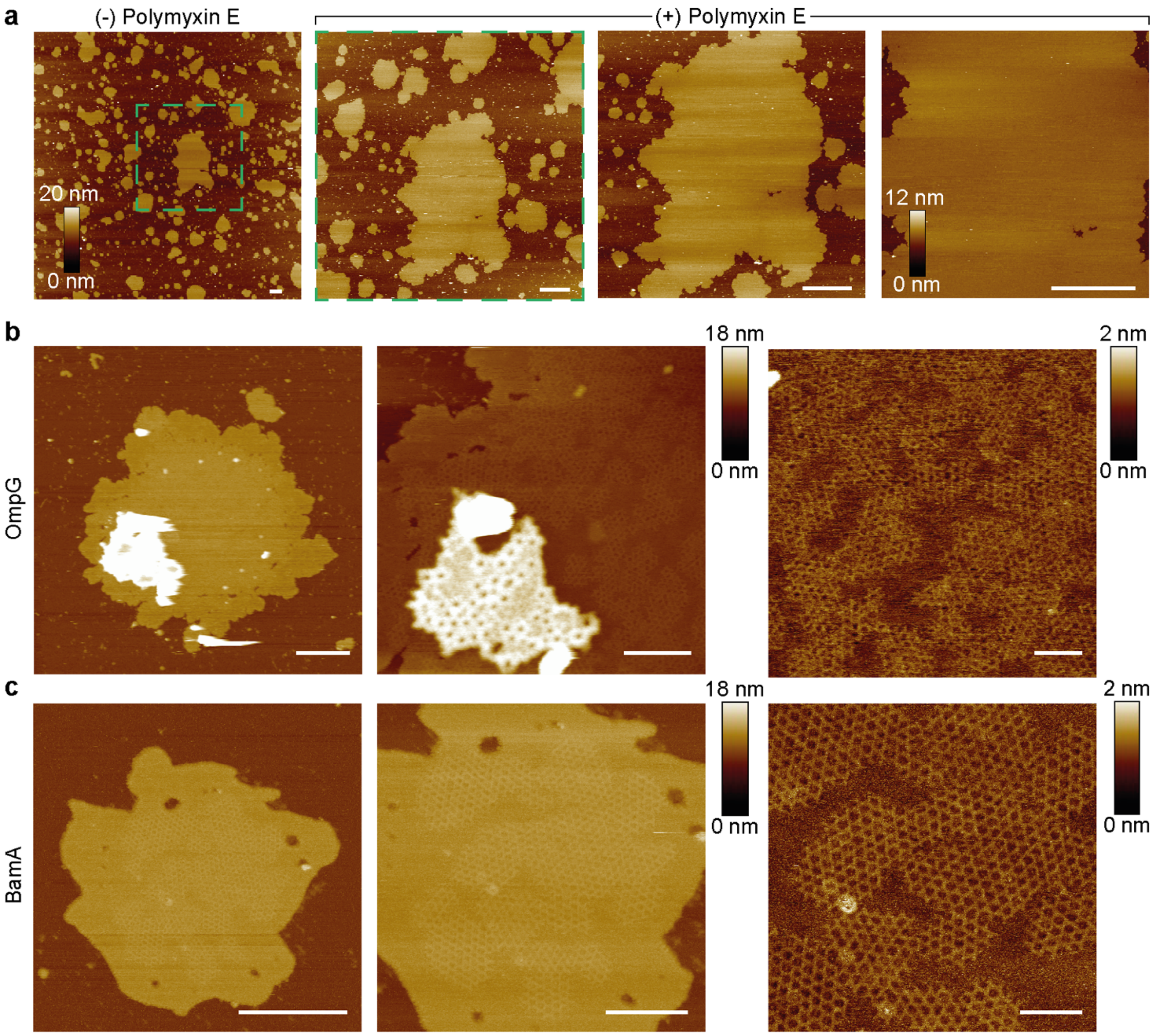

Supplementary Fig. 4. Formation of crystalline structures depends on the presence of LPS and is independent of over-expressed outer membrane proteins.

(a, b) AFM topographs of membrane patches produced from *E. coli* polar lipid (EPL) extract (Avanti Polar Lipids) vesicles, containing 67% PE, 23.2% PG, 9.8% CA, which are diluted in DPBS (1:100) and incubated with 60 mg/l polymyxin in DPBS buffer. Crystalline structures were not observed. Scale bars, 1μm. **(b, c)** AFM topographs of OM patches produced from BL21(DE3*) omp8 E. coli* strain overexpressing OmpG or BamA and after incubation with polymyxin in DPBS buffer. Scale bars, 200 nm (left), 100 nm (middle), and 50 nm (right). For a-c, each experiment was repeated independently three times with same results.

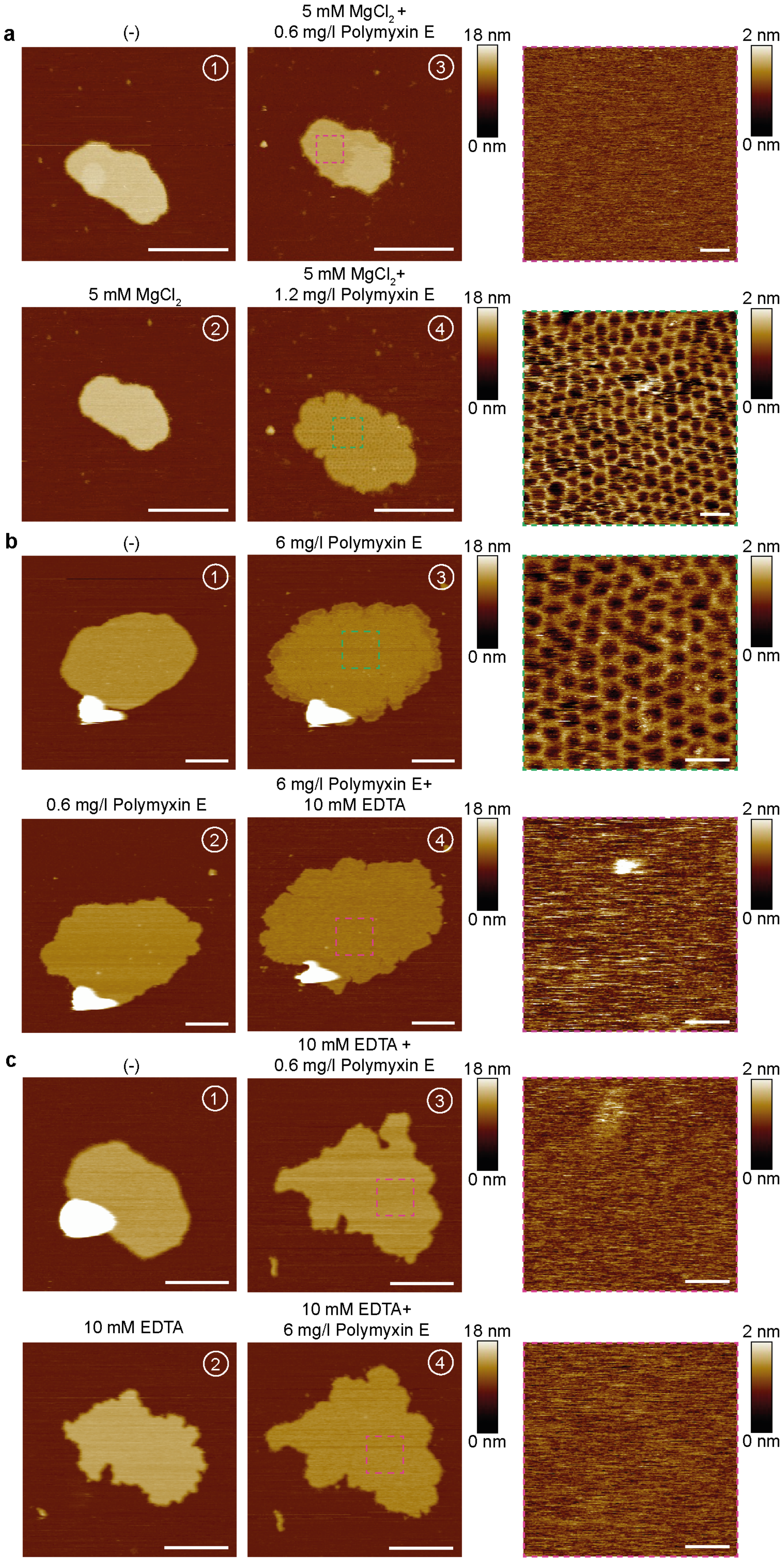
a
(-)
5 mM MgCl2 +
0.6 mg/l Polymyxin E
18 nm
0 nm
2 nm
0 nm
5 mM MgCl2
5 mM MgCl2+
1.2 mg/l Polymyxin E
b
(-)
6 mg/l Polymyxin E
0.6 mg/l Polymyxin E
6 mg/l Polymyxin E+
10 mM EDTA
c
(-)
10 mM EDTA +
0.6 mg/l Polymyxin E
10 mM EDTA
10 mM EDTA+
6 mg/l Polymyxin E
1
2
3
4

Supplementary Fig. 5. Dependence of crystalline structures on divalent cations.

(a-c) AFM topographs of OM patches from the *E. coli* MG1655 WT strain. **(a)** Crystalline structures occurred at higher concentrations of polymyxin treatment in the presence of 5 mM $MgCl_2$ supplemented DPBS buffer. Scale bars, 200 nm (left and middle) and 20 nm (right). **(b)** Ethylenediaminetetraacetic acid (EDTA), divalent cation chelating reagent, treatment abolishes the structures formed on OM patches upon polymyxin incubation (lower panel middle and right images). Scale bars, 200 nm (left, middle) and 20 nm (right). **(c)** Prior EDTA treatment of OM patches prevents the formation of crystalline structures on OM patches with polymyxin incubation (upper and lower panel middle and right images). Scale bars, 200 nm (left and middle) and 20 nm (right). For a-c, each experiment was repeated independently for three times with same results. Numbering in the images indicates the order of conditions scanned for each panel. (-) indicates conditions where AFM topographs were taken in DPBS buffer which is used as the base solution to prepare following conditions.

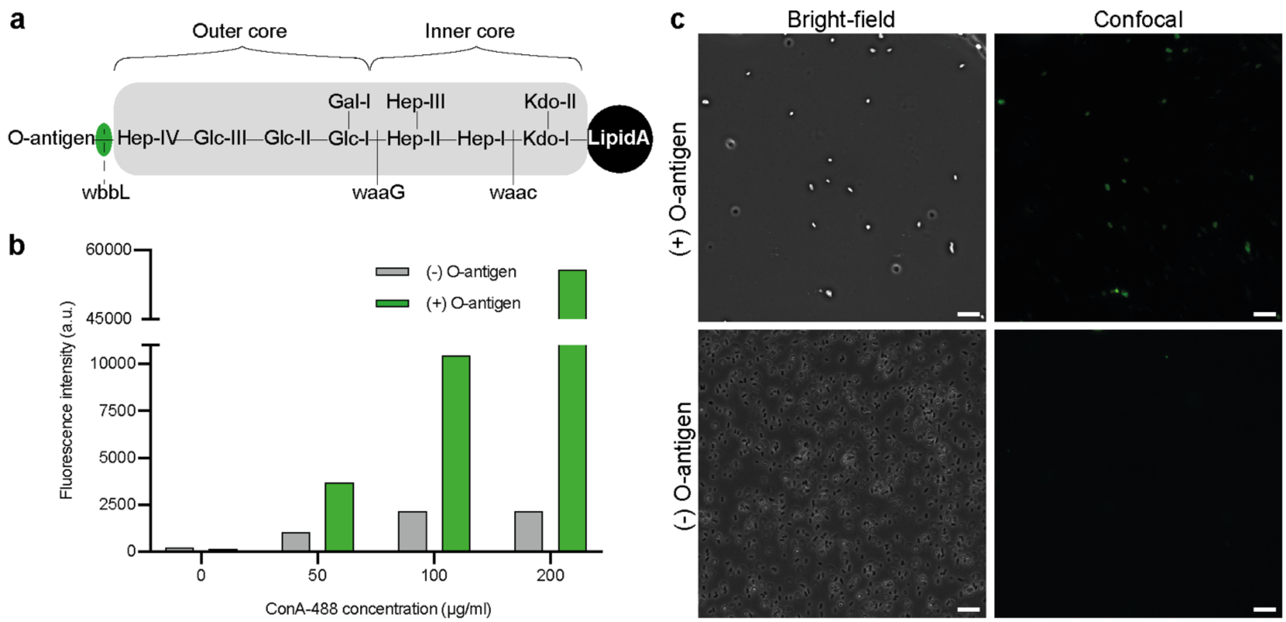

Supplementary Fig. 6. Restoration of O-antigen polysaccharide segment to the LPS of the *E. coli* MG1655 WT strain. **(a)** Schematic of smooth-type LPS structure[5]. Dashed lines indicate the linkages catalyzed by enzymes that take part in LPS biosynthesis. *wbbL* enzyme catalyzes the ligation of O-antigen to Hep-IV on LPS outer core. **(b)** Plate reader fluorescence assay data of O-antigen (+) and O-antigen (-) MG1655 strains incubated with increasing concentrations of Concanavalin A, Alexa Fluor-488 conjugate (ConA-488) – a lectin conjugate that specifically binds to O-antigen sugar residues. Fluorescence signal of O-antigen (+) MG1655 strain is considerably higher than O-antigen (-) MG1655 strain for all concentrations. Source data are provided as a Source Data file. **(c)** Detection of O-antigen with ConA-488 in cells with indicated genotypes. Bright-field and confocal images (green signal) of cells that were labelled with 200 µg/ml of ConA-488. The experiment was repeated independently two times with same results. Scale bars, 10 µm.

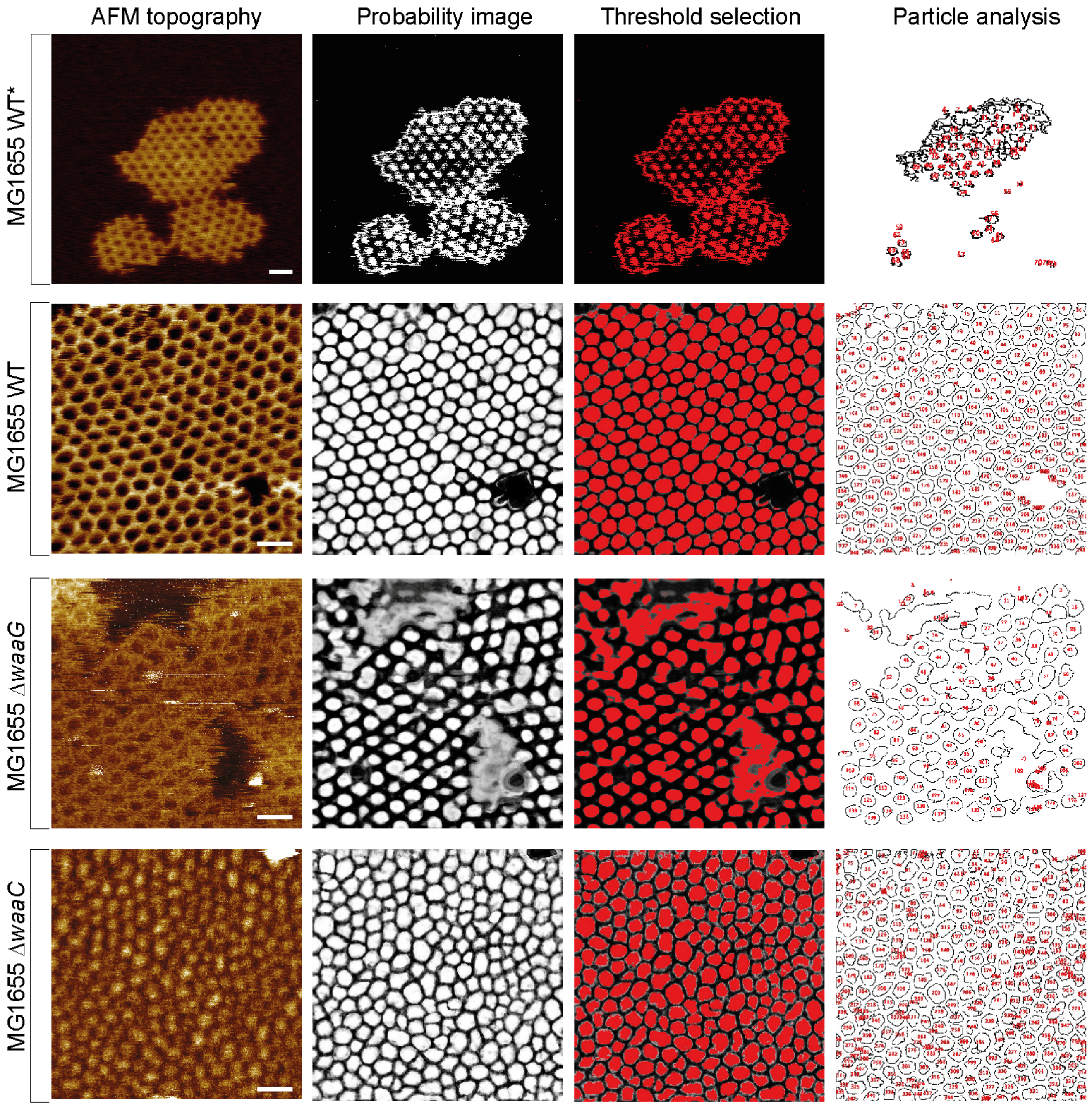

Supplementary Fig. 7. Diameter analysis for structures observed on polymyxin incubated OM patches produced from bacterial strains with varying LPS lengths.

Recorded AFM topographs of different *E. coli* strains are fed to ilastik software to generate probability images through pixel segmentation. To select the unit cells of crystalline structures Otsu thresholding was applied to the probability images using Fiji software. The diameter data of selected particles were further analyzed and filtered in Python using pandas library. For each bacterial strain, the experiment was repeated independently at least three times with similar results. Scale bars, 20 nm.

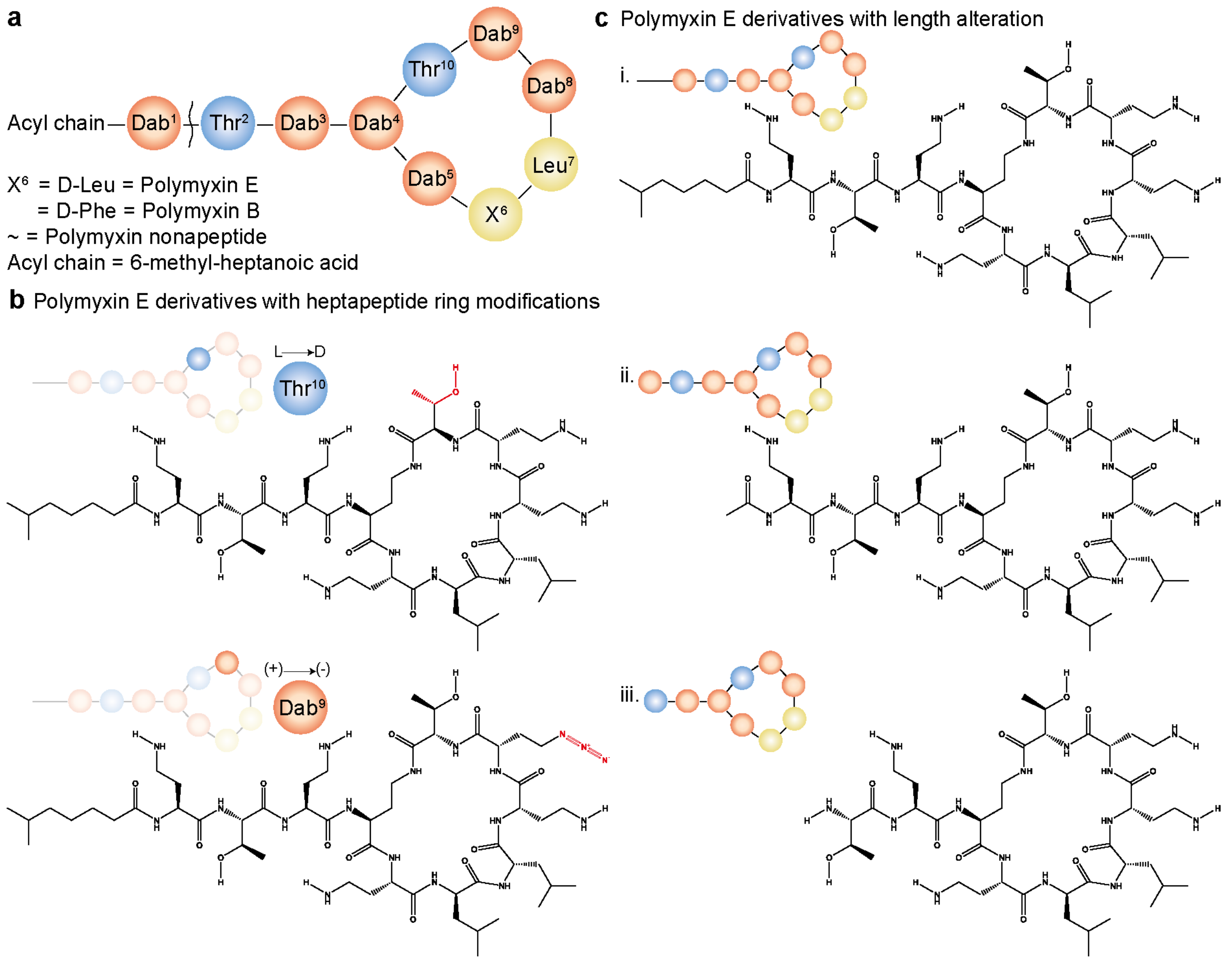

Supplementary Fig. 8. Chemical structures of the investigated polymyxin variants. (a) Schematic of polymyxin structure where cationic D-amino-butyric (Dab), polar threonine (Thr), hydrophobic leucine (Leu) and phenylalanine (Phe) residues are colored in orange, blue and yellow, respectively. **(b)** Chemical structures of polymyxin E derivatives with heptapeptide ring modifications, stereochemistry change in Thr^{10} (top) and charge change in Dab^{9} (bottom) residues. **(c)** Chemical structures of polymyxin E derivatives with length alterations from full (top) to intermediate (middle) and nonapeptide (bottom) *N*-termini.

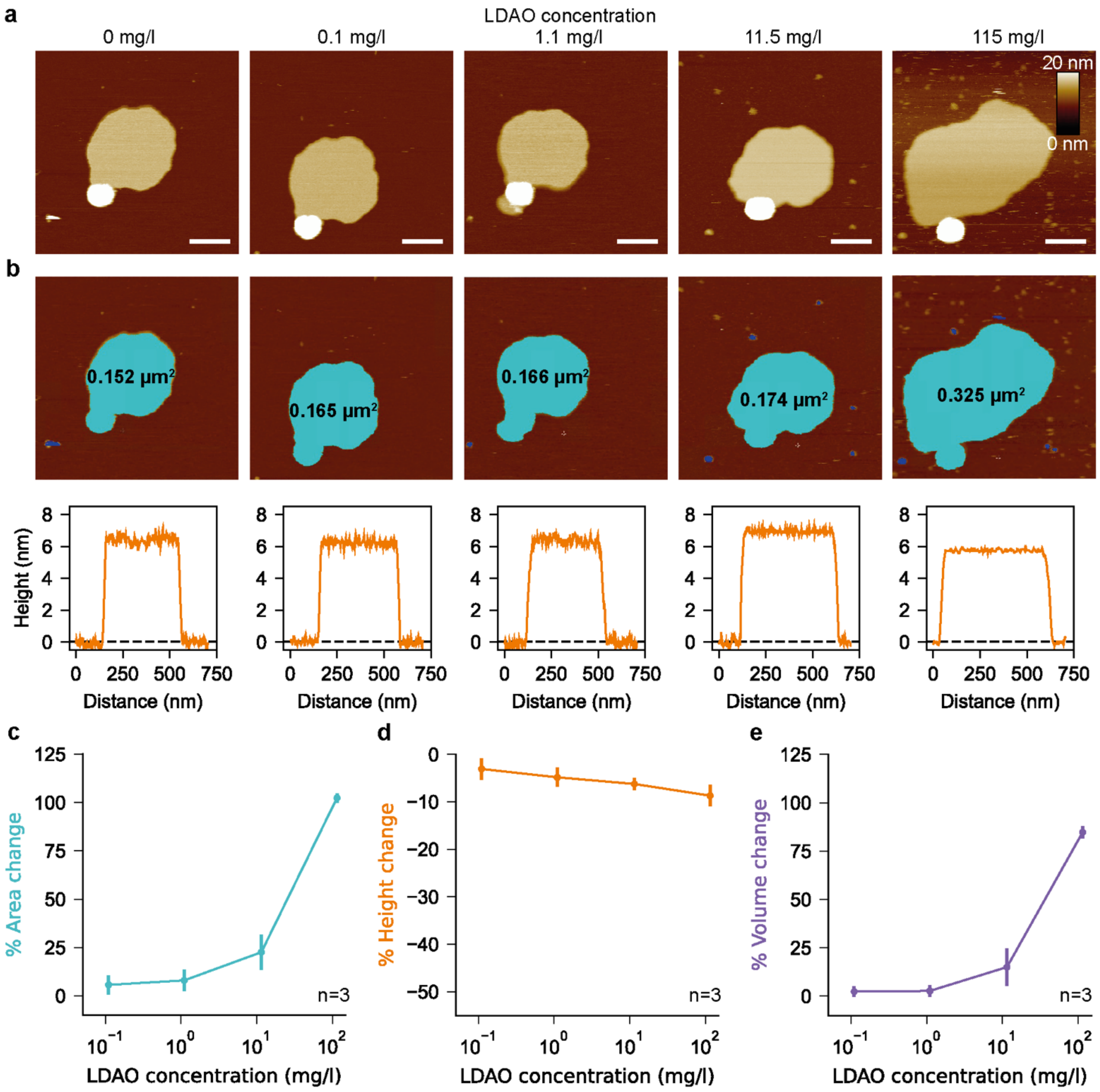

Supplementary Fig. 9. Effect of the common detergent LDAO on OM patches.

(a) AFM topographs of OM patches produced from the *E. coli* MG1655 WT strain are recorded for increasing concentrations of zwitterionic LDAO detergent in DPBS buffer. Scale bars, 200 nm. **(b)** Corresponding surface area (top) and height profiles (bottom) of patches are monitored at the same time. Treating OM patches (*n=3*) with increasing concentrations of LDAO considerably increases the **(c)** membrane area and slightly decreases the **(d)** membrane height, both of which effects increasing the **(e)** volume of the patch. In panels c-e, the error bars represent mean values +/- standard deviation (SD). Source data are provided as a Source Data file.

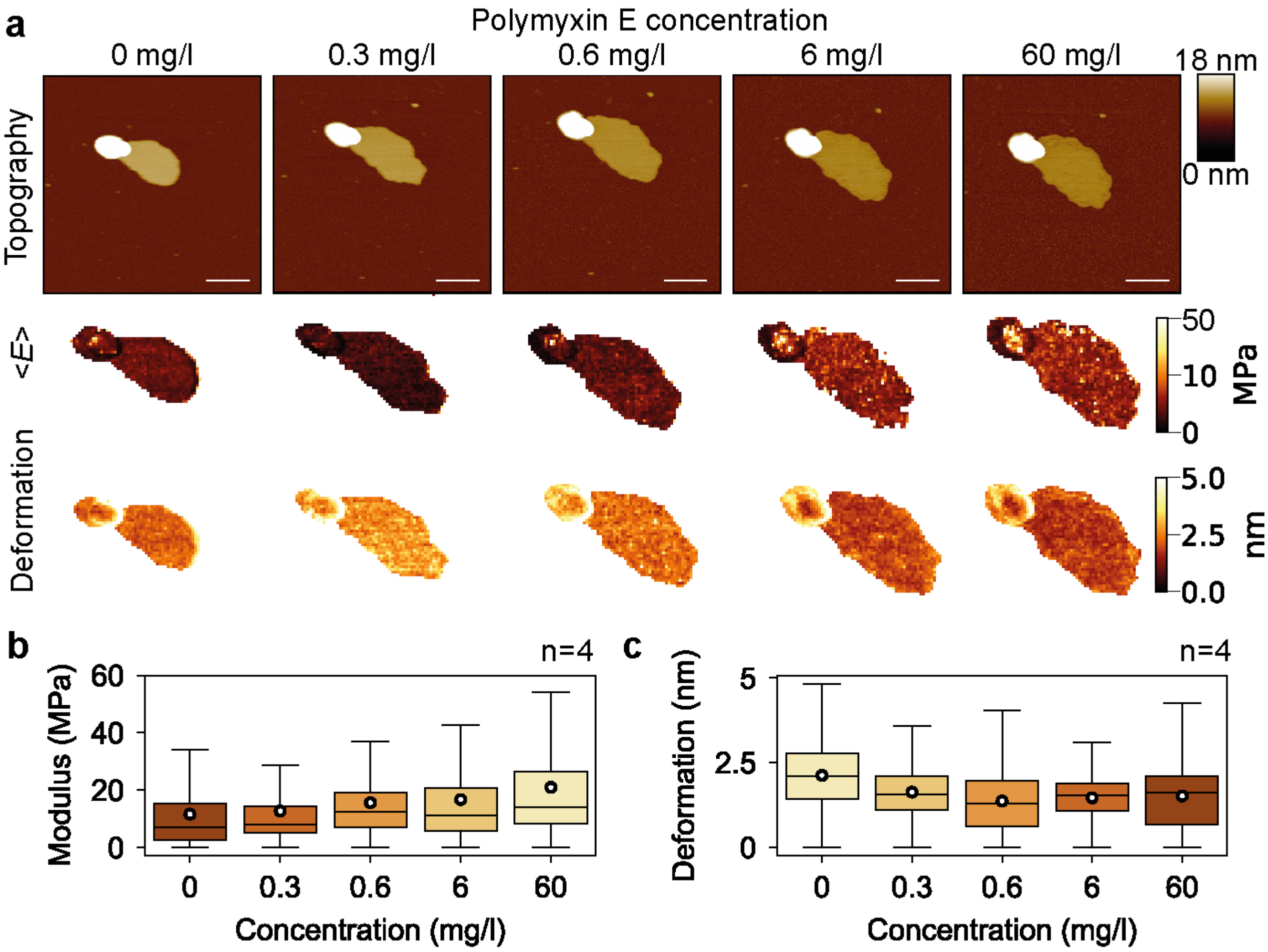

Supplementary Fig. 10. Formation of crystalline structures increases the elastic modulus and decreases the deformation of OM patches.

(a) AFM topographs (top), elastic modulus (middle), and deformation (bottom) maps of OM patches produced from the *E. coli* MG1655 WT strain recorded at increasing concentrations of polymyxin in DPBS buffer. The experiment was repeated independently four times with same results. Scale bars, 200 nm. **(b)** Average elastic modulus of OM patches increases with polymyxin concentration. **(c)** Average deformation of OM patches decreases with polymyxin concentration. For both experiments in panels **b** and **c**, all conditions are measured for *n*=4 OM patches. Circles represent the mean of a given condition. The lower and upper bound of a box indicates the 1st and 3rd quartile of the data, where the middle line is at the median (2nd quartile). The whiskers extending from the box show the range of the data. Source data are provided as a Source Data file.

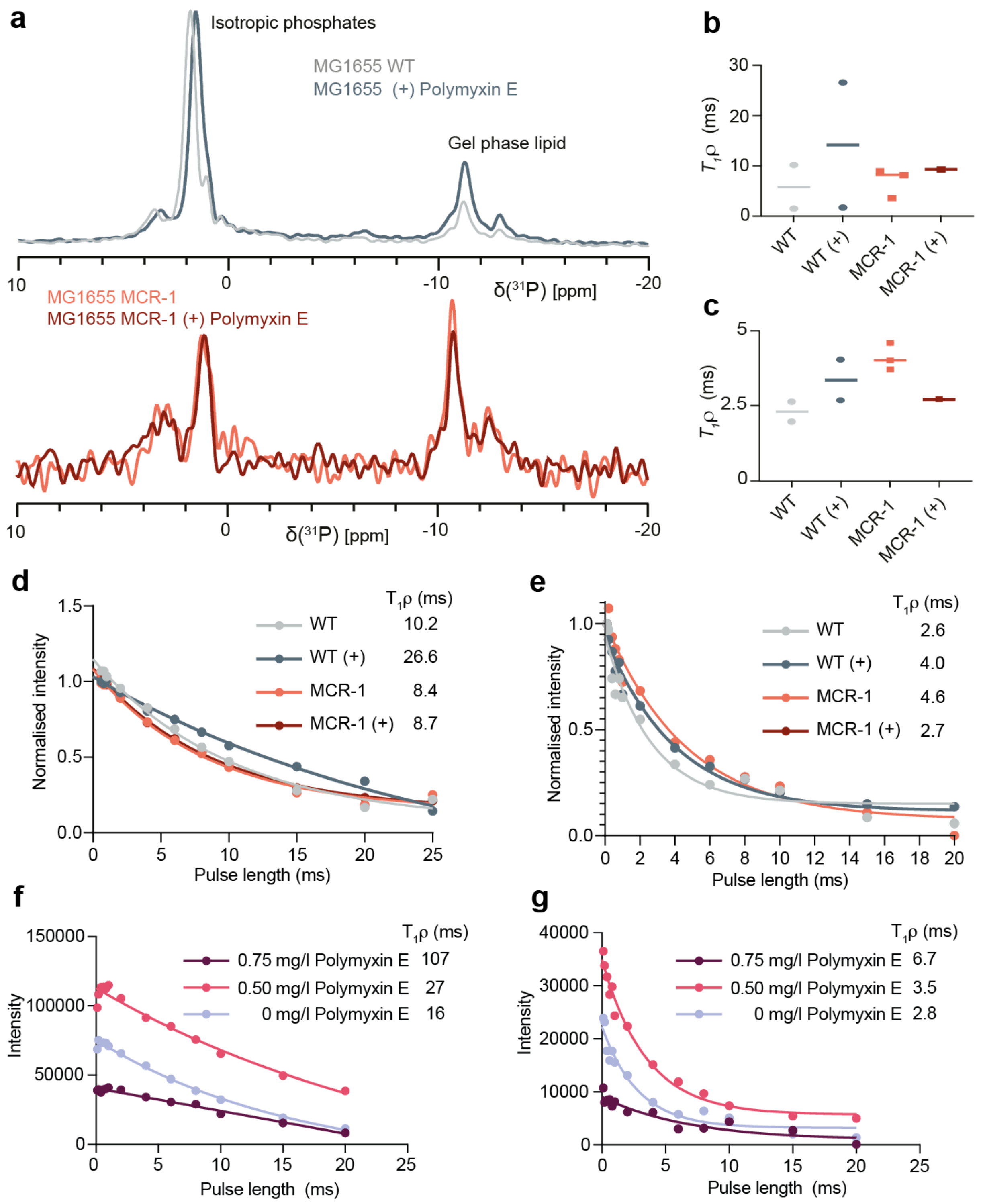

Supplementary Fig. 11. ^{31}P-NMR relaxation experiments on intact *E. coli* K-12 MG1655 cells.

(a) One dimensional ^{31}P-NMR spectra of the *E. coli* K-12 MG1655 WT and MCR-1 strain ± polymyxin E. **(b, c)** Longitudinal relaxation times in the rotating frame ($T_1\rho$) of (**b**) isotropic phosphates and (**c**) gel liquid phase determined by NMR relaxation experiments. Each dot represents the data from one experimental replicate, and the bar denotes the average. n = 1–3 independent experiments per condition. The negative control experiment was performed n=1 times, in full agreement with common practice in the field. **(d, e)** $T_1\rho$ relaxation experiments of WT and MCR-1 strains in the absence and presence of polymyxin E for (**d**) isotropic phosphates and (**e**) gel phase lipid. **(f, g)** $T_1\rho$ relaxation experiments of the (**f**) isotropic phosphates and (**e**) gel phase lipid for WT strain titrated with polymyxin E. Increased relaxation times with increasing polymyxin concentration indicate rigidification of the membranes.

Supplementary References

1. Maffei, E. et al. Systematic exploration of *Escherichia coli* phage–host interactions with the BASEL phage collection. PLOS Biology 19, 1–52 (2021).
2. Du, C. et al. Co-occurrence of the *mcr-1.1* and *mcr-3.7* genes in a multidrug-resistant *Escherichia coli* isolate from China. Infection and Drug Resistance 13, 3649–3655 (2020).
3. Prilipov, A., Phale, P. S., van Gelder, P., Rosenbusch, J. P. & Koebnik, R. Coupling site-directed mutagenesis with high-level expression: large scale production of mutant porins from *E. coli*. FEMS Microbiology Letters 163, 65–72 (1998).
4. Thoma, J. et al. Protein-enriched outer membrane vesicles as a native platform for outer membrane protein studies. Communications Biology 1, 1–9 (2018).
5. Bertani, B. & Ruiz, N. Function and biogenesis of lipopolysaccharides. EcoSal Plus 8, 1–33 (2018).
