# Supplementary material for: Antibiotic polymyxin arranges lipopolysaccharide into crystalline structures to solidify the bacterial membrane
Source: Nat Commun. 2022 Oct 21;13:6195. doi: 10.1038/s41467-022-33838-0 (PMC9587031; doi:10.1038/s41467-022-33838-0)
Supplement: Supplementary file 2 — Reporting Summary [file 41467_2022_33838_MOESM2_ESM.pdf]

nature portfolio

Corresponding author(s): Daniel J. Müller, Sebastian Hiller

Last updated by author(s): Aug 29, 2022

Reporting Summary

Nature Portfolio wishes to improve the reproducibility of the work that we publish. This form provides structure for consistency and transparency in reporting. For further information on Nature Portfolio policies, see our Editorial Policies and the Editorial Policy Checklist.

Statistics

For all statistical analyses, confirm that the following items are present in the figure legend, table legend, main text, or Methods section.

n/a	Confirmed	
☐	☒	The exact sample size (*n*) for each experimental group/condition, given as a discrete number and unit of measurement
☐	☒	A statement on whether measurements were taken from distinct samples or whether the same sample was measured repeatedly
☐	☒	The statistical test(s) used AND whether they are one- or two-sided<br>*Only common tests should be described solely by name; describe more complex techniques in the Methods section.*
☒	☐	A description of all covariates tested
☒	☐	A description of any assumptions or corrections, such as tests of normality and adjustment for multiple comparisons
☐	☒	A full description of the statistical parameters including central tendency (e.g. means) or other basic estimates (e.g. regression coefficient) AND variation (e.g. standard deviation) or associated estimates of uncertainty (e.g. confidence intervals)
☐	☒	For null hypothesis testing, the test statistic (e.g. *F*, *t*, *r*) with confidence intervals, effect sizes, degrees of freedom and *P* value noted<br>*Give P values as exact values whenever suitable.*
☒	☐	For Bayesian analysis, information on the choice of priors and Markov chain Monte Carlo settings
☒	☐	For hierarchical and complex designs, identification of the appropriate level for tests and full reporting of outcomes
☒	☐	Estimates of effect sizes (e.g. Cohen's *d*, Pearson's *r*), indicating how they were calculated

Our web collection on statistics for biologists contains articles on many of the points above.

Software and code

Policy information about availability of computer code

Data collection	All commercial software used for collecting the presented data (AFM, confocal imaging, multi-well growth assay, cryo-EM, FACS, and solid-state NMR) is stated with their versions in the Methods section of the manuscript.<br>AFM: Nanoscope v.1.8<br>Confocal imaging: Nikon Eclipse Ti2-E<br>Multi-well growth assay: Tecan EVO 200 and Tecan Infinite M200 Pro<br>Cryo-EM: FEI Talos TEM<br>FACS: BD FACSAria III<br>Solid-state NMR: Bruker 11.7 Tesla NMR spectrometer
Data analysis	Data analysis and visualization scripts used in this study are publicly available in the Zenodo repository, https://zenodo.org/badge/latestdoi/444965043, and is publicly available as of the date of publication (MuellerLabETHZ, 2022).

For manuscripts utilizing custom algorithms or software that are central to the research but not yet described in published literature, software must be made available to editors and reviewers. We strongly encourage code deposition in a community repository (e.g. GitHub). See the Nature Portfolio guidelines for submitting code & software for further information.

Data

Policy information about availability of data

All manuscripts must include a data availability statement. This statement should provide the following information, where applicable:

- Accession codes, unique identifiers, or web links for publicly available datasets
- A description of any restrictions on data availability
- For clinical datasets or third party data, please ensure that the statement adheres to our policy

The experimental data that support the findings of this study is available within this article or its supplementary materials. The datasets for lattice constant, biophysical change and modulus map analysis are available in the Zenodo repository, https://zenodo.org/badge/latestdoi/444965043, and is publicly available as of the date of publication (MuellerLabETHZ, 2022). Any additional information required to reanalyze the data reported in this paper will be shared by the corresponding authors upon request.

Human research participants

Policy information about studies involving human research participants and Sex and Gender in Research.

Reporting on sex and gender	N/A
Population characteristics	N/A
Recruitment	N/A
Ethics oversight	N/A

Note that full information on the approval of the study protocol must also be provided in the manuscript.

Field-specific reporting

Please select the one below that is the best fit for your research. If you are not sure, read the appropriate sections before making your selection.

☒ Life sciences ☐ Behavioural & social sciences ☐ Ecological, evolutionary & environmental sciences

For a reference copy of the document with all sections, see nature.com/documents/nr-reporting-summary-flat.pdf

Life sciences study design

All studies must disclose on these points even when the disclosure is negative.

Sample size	Sample sizes were not predetermined based on statistical methods, but were chosen according to the standards of the field (three or more independent biological replicates for each condition, or a titration series, or at least single experiments for relaxation time measurements).
Data exclusions	Data were not excluded from analysis.
Replication	Reported results were consistently replicated across multiple experiments where all replicates generating similar results. The number of experiments is specified in each Figure caption.
Randomization	No randomization was necessary for this study, because all experimental parameters were controlled, as described. Randomization is not generally used in this field.
Blinding	Investigators were not blinded. Blinding during collection was not needed because conditions were well controlled. Blinding during analysis was not feasible because the results were quantitative and did not require subjective judgment or interpretation. Blinding is not typically used in the field.

Reporting for specific materials, systems and methods

We require information from authors about some types of materials, experimental systems and methods used in many studies. Here, indicate whether each material, system or method listed is relevant to your study. If you are not sure if a list item applies to your research, read the appropriate section before selecting a response.

Materials & experimental systems

n/a	Involved in the study
x	[ ] Antibodies
x	[ ] Eukaryotic cell lines
x	[ ] Palaeontology and archaeology
x	[ ] Animals and other organisms
x	[ ] Clinical data
x	[ ] Dual use research of concern

Methods

n/a	Involved in the study
x	[ ] ChIP-seq
x	[ ] Flow cytometry
x	[ ] MRI-based neuroimaging
