# Supplementary figures and images for: Antibiotic polymyxin arranges lipopolysaccharide into crystalline structures to solidify the bacterial membrane
Source: Nat Commun. 2022 Oct 21;13:6195. doi: 10.1038/s41467-022-33838-0 (PMC9587031; doi:10.1038/s41467-022-33838-0)

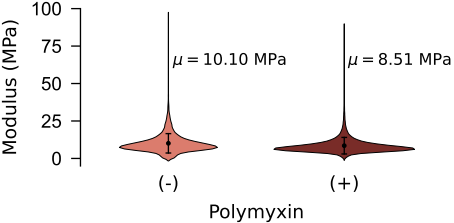

Supplement: Supplementary file 3 — Source Data [file 41467_2022_33838_MOESM3_ESM.zip › Source_Data/Fig6a-b/MCR1/MCR_Modulus_Main.pdf]

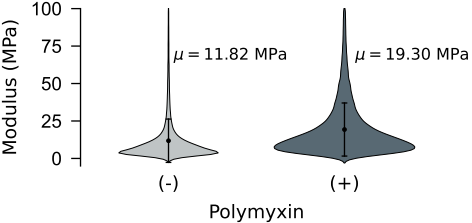

Supplement: Supplementary file 3 — Source Data [file 41467_2022_33838_MOESM3_ESM.zip › Source_Data/Fig6a-b/WT/WT_Modulus_Main.pdf]
